# Wild thyme (*Thymus serpyllum* L.): a review of the current evidence of nutritional and preventive health benefits*

**DOI:** 10.3389/fnut.2024.1380962

**Published:** 2024-05-23

**Authors:** Banaz Jalil, Ivo Pischel, Björn Feistel, Cynthia Suarez, Andressa Blainski, Ralf Spreemann, René Roth-Ehrang, Michael Heinrich

**Affiliations:** ^1^Pharmacognosy and Phytotherapy, UCL School of Pharmacy, London, United Kingdom; ^2^Dr. Ivo Pischel Consulting, Rossbach, Germany; ^3^Finzelberg GmbH & Co. KG, Andernach, Germany; ^4^Chinese Medicine Research Center, Department of Pharmaceutical Sciences and Chinese Medicine Resources, College of Chinese Medicine, China Medical University, Taichung, Taiwan

**Keywords:** wild thyme, *Thymus serpyllum*, nutrition, health prevention, phytopharmacology, traditional use, gut-brain interaction, rosmarinic acid

## Abstract

*Thymus serpyllum* L. (Lamiaceae), known in English as ‘wild thyme’, is primarily found in the Palearctic realm (Eurasia, North Africa) and has been utilized traditionally for culinary, nutritional, medicinal, and aromatic purposes. The essential oil extracted from wild thyme is particularly noteworthy, being used extensively in the food industry as a flavoring agent and preservative. The plant’s aerial parts are commonly employed as an element of the diet (e.g., tea)/for culinary uses and in local/traditional medicine (primarily for managing respiratory and gastrointestinal conditions), similar to the use of common thyme. There is practically no information available on the species’ nutritional benefits. Pharmacological studies, including *in vitro* and *in vivo* research, alongside a limited number of clinical trials, have investigated extracts of *Thymus serpyllum*, although these extracts are often phytochemically poorly characterized in different experimental protocols and models. These studies have demonstrated a range of therapeutic effects, such as antimicrobial (notably the essential oil) and anti-inflammatory, as well as its preventative health benefits and nutritional value of wild thyme. Preclinical studies have corroborated the plant’s anti-inflammatory potential, particularly in conditions like inflammatory bowel diseases (IBD) and irritable bowel syndromes (IBS). Additionally, evidence of hepatoprotective activities and benefits in managing metabolic syndrome and cardiovascular health issues, such as lipid metabolism regulation, cholesterol reduction, antidiabetic, antihypertensive, and immunomodulatory effects, have been observed predominantly in rodent models. Phytochemical analysis of wild thyme reveals an essential oil fraction below 1%, along with non-volatile compounds predominantly comprising phenolic acids (such as rosmarinic, salvianolic, and caffeic acids) and flavonoids (mainly glucosides of luteolin, apigenin, and their derivatives). These components are believed to contribute significantly to the plant’s medicinal, nutritional, and preventive health properties. Despite promising findings, there is a need for more rigorously designed controlled clinical trials using phytochemically characterized wild thyme. The plant has an excellent safety and tolerability record. This review at the interface of nutritional/preventive health properties and as pharmacological activities highlights the current role of wild thyme in nutrition and general healthcare as well as its future potential, and also points to important gaps in the literature.

## 1 Introduction

*Thymus serpyllum* L. (Lamiaceae), commonly known as wild thyme, presents a compelling case study in the field of ethnobotany, nutrition and phytotherapy. This perennial herb thrives in the diverse climates of the Palearctic realm, although nowadays cultivated in many world regions for its aromatic essential oil or the herbal part used in food or as medicine in the food and pharmaceutical industry (1). So far, a limited number of reviews on *Thymus serpyllum* (*T. serpyllum*) have been conducted mainly on the characteristics and properties of the essential oil for its antimicrobial, antioxidants, and anti-inflammatory activities (2–4) whereas a monograph about the genus Thymus, including wild thyme, was published two decades ago (1). This review endeavors to unravel the complexities of wild thyme, providing an in-depth exploration of its botanical attributes, traditional applications, nutritional value, pharmacological potential, and safety aspects.

The cultural and historical context of wild thyme is as rich and varied as its pharmacological portfolio. Traditionally appreciated for its role in herbal teas and as a culinary enhancer, particularly for its aromatic essential oil, *T. serpyllum* has long been a staple in various regional cuisines and medicinal practices (1). The use of its aerial parts in traditional medicine, especially for respiratory and gastrointestinal complaints, underscores its significance in ethnomedicine.

In recent years, the scientific inquiry has shifted toward understanding the phytochemical composition of wild thyme. The presence of essential oils, phenolic acids, and flavonoids in the plant points to potentially complex phytochemical synergies underpinning its preventive health benefits and therapeutic properties (1). These compounds have been the focus of various *in-vitro* and *in-vivo* studies, along with clinical trials, which collectively suggest promising antimicrobial, anti-inflammatory, hepatoprotective, and cardio-metabolic effects. The plant’s benefits in managing conditions like inflammatory bowel diseases and irritable bowel syndromes, as well as its positive impact on the gut-brain axis and the gut microbiome, are particularly noteworthy.

Despite its established traditional use and emerging scientific backing, there remains a gap in the form of well-designed controlled clinical trials. This review aims to highlight the importance of such studies to further assess the safety, nutritional value, and efficacy of *T. serpyllum*. By bridging traditional knowledge with scientific research, this review seeks to contribute to understanding wild thyme’s role in modern herbal medicine and stimulate further research in this promising field.

## 2 Methodology

### 2.1 Search strategy

**Figure 1:**
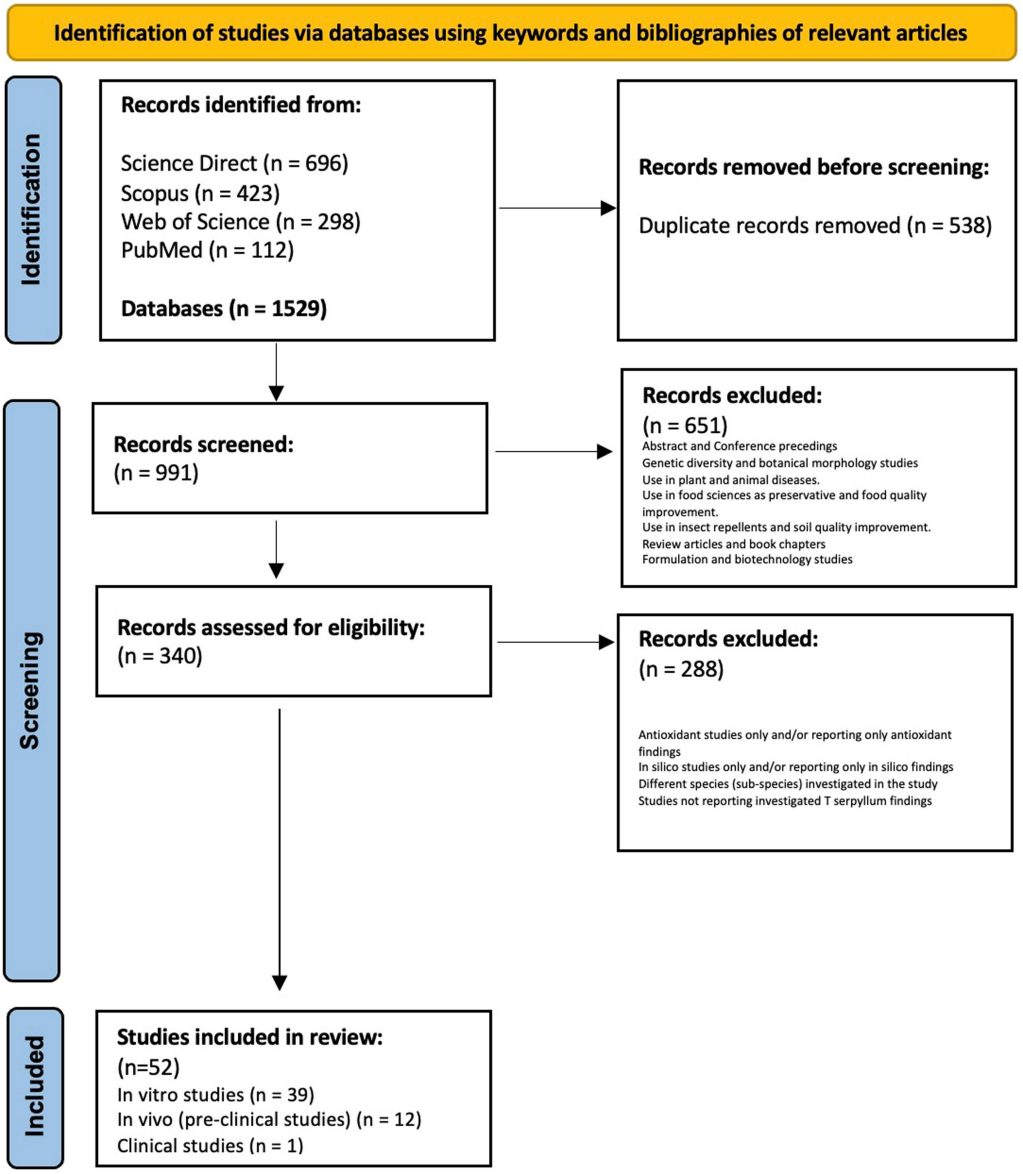
Workflow chart for inclusion and exclusion of studies in the systematic review using PRISMA model (5).

A systematic literature search was performed according to the PRISMA guidelines (5). The main databases included were Web of Science (core collection), PubMed, Scopus, and ScienceDirect, covering the period from the inception to December 2023. Articles published in several languages, mainly English, French, and German (i.e., articles without country or language restrictions), were reviewed. The terms searched with their combinations were *Thymus serpyllum*, wild thyme, chemical constituents, phytochemistry, biological activity, nutrition, pharmacology, toxicology, phytopharmacology, and traditional use. Additional information was retrieved by manually searching the studies and review articles. All articles were then exported to Rayyan software for review and evaluation. The workflow of the search strategy is shown in Figure 1. In addition, to understand the role of *T. serpyllum* in modern functional food and dietary supplement markets, we conducted a comprehensive search of relevant sub-categories through the Mintel database. Specifically, the search covered “Sugar & Gum Confectionery” and “Sauces & Seasonings” as well as “Personal Insect Treatment/Repellents,” “Decongestants, Cough, Cold & Flu Relief” and “Vitamins & Dietary Supplements.” The search yielded a total of 65 hits, indicating the growing interest in and use of *T. serpyllum* in various consumer product categories.

### 2.2 Study selection criteria

The literature search was conducted by two of the authors (BJ, IP), and next, the identified titles, abstracts and studies were evaluated, with any differences resolved through discussions among the authors of this review. The inclusion criteria that the review focuses on *T. serpyllum* L., including its chemistry, nutrition, pharmacology, toxicology and clinical assessment, with clear evidence that *T. serpyllum* and no other species of the genus were used. The studies, including the experimental approaches, phytochemical characterization and reporting of the plant materials and their initial processing, were assessed and evaluated following Heinrich et al. (6); Heinrich et al. (7). Specifically, the extracts/extraction process, dosage, control, and experimental models, including methodological details and reported outcomes were assessed (Supplementary Tables S2, S3). Studies using chemical antioxidant assays with claims about pharmacological activity were excluded since there is no evidence for therapeutic benefits on the basis of such chemical assays and many journals, e.g., in the field of food sciences and pharmacology, no longer accept them as relevant assays but only for chemical-analytical purposes (6).

### 2.3 Patent search strategy

A systematic patent search was performed by two of the authors (BF, IP) in several patent databases, including Espacenet, USPTO, Google Patents and Orbit.com. Two different search strategies were used, using different search terms, strings and several filters. The four patent databases used are FAMPAT: https://orbit.com/; GOOGLE PATENTS: https://patents.google.com/; ESPACENET: https://worldwide.espacenet.com/patent/search; USPTO: https://ppubs.uspto.gov/pubwebapp/. In the first strategy, the commercial Orbit’s patent database Fampat (The Fampat Collection; orbit.com) was used. Fampat combines results from “EP and PCT publications and links between US provisional and published applications” within a database-specific search strategy and language to search specifically for certain patent classes. Here, the important patent class – the IPC A61 patent class and all subclasses for ‘Medical and Veterinary Science, Hygiene’ use – including medicinal teas – was applied. Furthermore, only ‘thymi’ and ‘serpylli’ were used, which automatically includes translations such as thyme or thymus or serpyl or serpolet were used. This query returned 71 hits, five of which are relevant in the scope of this review (Supplementary Table S1).

In the second arm of our strategy, the open access patent databases Google Patents, Espacenet and USPTO were used. The main search term was “*thymus serpyllum*” combined with specific classification categories (IPC a/o CPC), e. g.: A61K36/53: IPC for Lamiaceae, A61K2236/30: IPC for extract/ion, and A23F3/16: IPC class for Tea extraction; Tea extracts; Treating tea extract; preparation of instant tea. This approach eventually resulted in a reasonable number of hits, less than 100, which were evaluated in more detail. After checking for country-specific multiple entries, the duplicates were removed. Concerning relevance, ten documents remained (see Table 1; Supplementary Table S1).

**Table 1:** Patent details of the relevant *T. serpyllum* patents, ordered by priority date (for granted patents the patent number is in bold).

Pat#	Relevance re Ts in: TI/AB/CL/TB	Priority date	Patent number	Title	Assignee/Inventor/s	Abstract (AB; title & details)
1	Medium AB/CL/TB	1990-02-28	**RO98315B1**	Medicinal tea	Ernest Macalik; Ludovic Scocs; Stefan Vandor	Medicinal tea with 9 plants for gastro-intestinal complaints; containing 10% *Thymus serpyllum*
2	Medium AB/CL/TB	1990-02-28	**RO98319B1**	Vegetable water extract	Elemer Zagoni; Ernest Macalik; Stefan Vandor	Aqueous vegetable extract which consists of 15% of Ts herba plus 6 other plants (tea blend) used for aerosol therapy in bronchial complaints
3	Medium AB/CL/TB	2009-03-13	RO125695A2	Phytopharmaceutical product with effects upon the respiratory tract	Mihai Codrut Tutu; Zvezomir Hadjiev-Marinov	Phytopharmaceutical product (alc. fluid) used as a dietary supplement in lung and respiratory tract disease obtained by macerating in 25–30% alcohol & 6 plants +Ts + Tv (herbal mix)
4	Low TB	2009-04-23	WO2009049900A1 (US20100240768A1)	Novel nutraceutical compositions containing thymol and/or p-cymene or plant extracts for cognition	Fowler, Ann; Goralczyk, Regina; Kilpert, Claus; et al.	Nutraceutical composition (essential oils or plant extracts) containing thymol and/or p-cymene useful for improvement of cognitive functions, psycho-social status, such as learning, memory alertness, psychotic stability, maintenance.
5	Low TB: Ts within a plant list	2010-09-06	WO2012033422A1	Medical cosmetic herbal composition on the basis of herbal mixture and the procedure for its obtention	Aleksandar Pavlov, Jovanka Bubnjevic – Pavlov	TI & as a spray, based on herbal extracts (ethanol or glycerol plus water), for treating diseases, disorders of facial, body skin etc.
6	High: TI/AB/CL/TB	2013-12-12	WO2013182709A1**(US11179430B2)**	Extracts from Mother of thyme and the use thereof	Bernd Walbroel, Ivo Pischel, Björn Feistel	Extracts of *T. serpyllum* for use in (inflammatory) gastro-intestinal ailments; deals only with *T. serpyllum*
7	Low TB (within a plant list)	2014-02-28	US20230043427A1	Composition For Making a Tea Beverage or Herbal and Vegetable Broths	Philippe Ragot; Bernard Mompon; Cedric Rousseau; et al.	infusion product for (tea) beverages, with fruits, herbs, medicinal plants, tea, vegetables and/or spices
8	Medium CL/TB	2014-10-09	CN104305394A	Instant drink comprising bubbles and dissolved gas and drink system	Zhang Biyun	TI & (hot, cold) drinks, with tea, fruit & vegetable juice, condiment, nutritional ingredients
9	Low TB: Ts within a long plant list	2015-08-26	WO2016030409A1	Method for making reconstituted plant material using extrusion or molding processes and products so obtained	Cedric Rousseau	TI & plant material with fibers, extracts, substances, for an edible product.
10	Low TB: Ts within a long plant list	2016-02-25	WO2016139561A1 (US20160255854A1)	Low bulk density composition for making a tea beverage having reduced dust or fines	Cedric Rousseau	TI & plant materials (extract, concentrate) formed into a fibrous structure or network, such as a sheet or fibrous layer, with minimal fines, dust, low bulk density.
11	Medium CL/TB	2016-09-26	CN106344648B	Thymus plant, extract and application thereof	Chen Xiaoyi; Bai Shaojuan; Zhao Yicheng; et al.	thymus plants (Ts within list of (Asian) T. spp.) preparation with 1. acidified acetone, 2. ethyl acetate; containing carvacrol, tanshinol, scutellarein, 5, 4′ -dihydroxy-6, 7, 8-trimethoxyflavone, oleanolic acid, rosmarinic acid; TCM for treating cardio- & cerebrovascular diseases.
12	Low TB: Ts within a long plant list	2016-11-02	US2022354919A1	Extract of an herbal composition as antimicrobial and/or antibiofilm agent	Laure Breulis; Pascal Mayer	TI & synergistic bioactives, for nutraceutical, pharmaceutical or cosmetic use
13	Medium AB/CL/TB	2016-07-14	CN106139077A	A kind of fragrant plant hair growth promoter and preparation method thereof	Yu Hanmou; Jiang Xingtao; Wang Xiaoli	TI & 7 plant extracts plus Ts extract 0.5–2%, plant base oil 75–90%, antioxidant 0.05–0.1%; activate hair follicle etc. of scalp, cells, is safe, nontoxic, simple preparation
14	Low TB: Ts within list of (Asian/TCM) T. spp.	2016-07-04	CN106109564A	Pharmaceutical composition for the treatment of cerebral thrombosis and preparation method thereof	Pang Dakun; Kong Qianqian	TI & based on several plants incl. Herba thymi vulgaris
15	Low TB: Ts within list of (Asian/TCM) T. spp.	2021-02-15	CN112957391A	Thymus plant extract, preparation method and application	Mei Yanxin; Wang Xiuhuan	TI & based on thymus plants, for liver injury, protection, treatment

TI, AB, CL & TB: title, abstract, claims and text body (of an IP document); Ts, *T. serpyllum*; Tv, *T. vulgaris*.

## 3 Results and discussions

### 3.1 Botanical description and morphology

**Figure 2:**
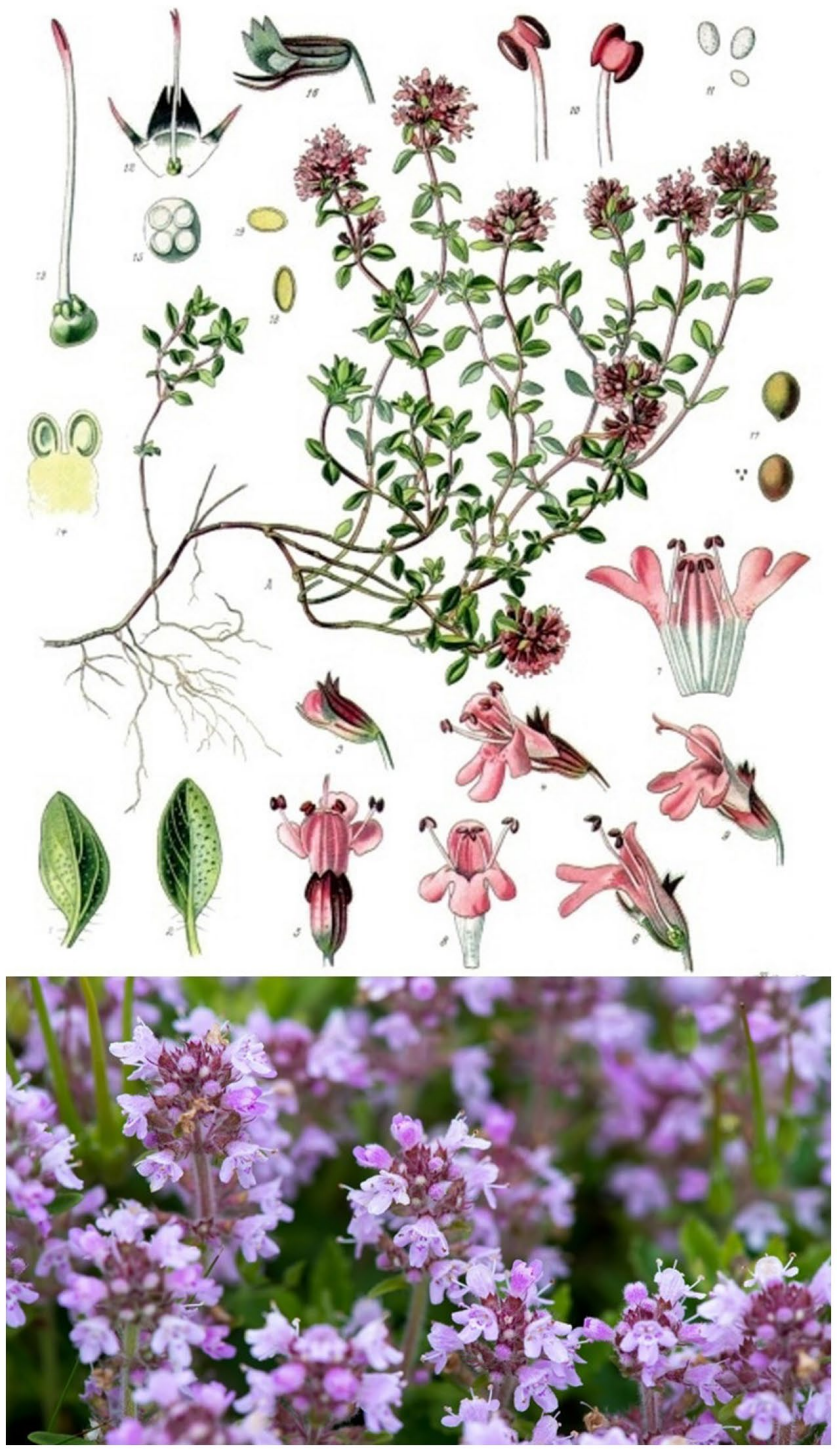
Botanical drawing (top) and flowering wild thyme (bottom) of *Thymus serpyllum* (10), licenses acquired of botanical drawing (top) by Alamy (11), and flowering wild thyme (bottom) by 123RF (12).

Wild thyme is an evergreen, ground-covering subshrub that reaches heights of 2 to 10 cm. It is forming a mat with closely prostate runners, which become woody (8). These lying stems are long, creeping, continue to grow for years and usually end with a tuft of leaves, rarely with an inflorescence. The branches are always hairy all around. The leaves are linear to narrowly elliptical or obovate, 3 to 10 mm long, 1 to 3 mm wide and short-stalked or sessile. The flower shoots hardly differ in size and shape. The uppermost pair of lateral nerves is usually lost and does not unite to form a peripheral nerve. The leaf blade is ciliated in the lower third, as is the petiole. The blade is rarely hairy. Their lateral nerves are blunt on the underside. The flowering period is from July to September. The inflorescence is capitate, faintly aromatic when fresh (8). The hermaphrodite flowers are zygomorphic and fivefold with a double perianth. The calyx is about 3.5 to 4.5 mm long, hairy below and glabrous above and imperforate at the teeth. The upper calyx teeth are broadly triangular and about as long as they are wide at the base (8, 9). The cytology reveals that the diploidic number of chromosomes is 2n = 24 (8, 9) (Figure 2).

#### 3.1.1 Botanical name, etymology of the plant name, and common names in various languages

The botanical binomial name of wild thyme is *Thymus serpyllum* L., with many botanical synonyms due to numerous descriptions of several authors depending on different regions and occurrences of the plant. Many synonyms are tabularly compiled in the literature and databases (13). Different literature sources give reasonable explanations for the etymology of plant names “thymus” and “serpyllum.” For the genus name “Thymus,” there seem to be two possible ancient Greek origins: 1. θύμον (thúmon) from θύω (thúō, “to smoke”) (14) or θῡμῐ́ᾱμᾰ (thūmíāma), meaning “that which is burnt as incense or fumigation” for its fragrance, likely because of its use in religious celebrations or temple services. The latter is at least described several times in Herodotus’ Histories (15). Secondly, the word could stem from thumos, which means “courage or strength” (16), where thyme symbolized bravery, perhaps due to the refreshing and powerful scent of wild thyme, although confusingly “thymos” (lat. Thymus) also refers to the thymus gland. The descriptive specific epithet (species name) “serpyllum” is also of Greek origin ἕρπυλλος (hérpullos), from ἕρπω (hérpō, “to creep, crawl”), thus, meaning “creeping.” In Latin, it became serpere, i.e., “to creep.” Wild thyme is also called Creeping Thyme (17). Based upon this Greek/Latin origin, various regional names, botanical synonyms and common names in different languages derived. Thus, the English name is wild or creeping thyme, mother of thyme, Breckland thyme; the German name is Quendel, Sand-/Feldthymian; in French Serpolet, Italian Serpillo, Spanish Tomillo silvestre/serpoleto, Portuguese Serpão, Tomilho serpão/selvagem, Polish Macierzanka piaskowa, Serbic Душичка, Мајчина душица, Материна душица/Dušička, Majčina dušica, Materina dušica, Bulgarian Мащерка дива/Masherka diva, Swedish Backtimjan, Hindi: Banajwain, Chinese: 蒙百里香, Japanese: ボウシュウボク, Korean: 세르필룸백리향, whereas the (Phyto)Pharmaceutical terms are: Herba Thymi serpylli, Herba serpyllum, Serpylli herba (18).

### 3.2 Ecology and cultivation

#### 3.2.1 Maps of global and world distribution of *Thymus serpyllum*

**Figure 3:**
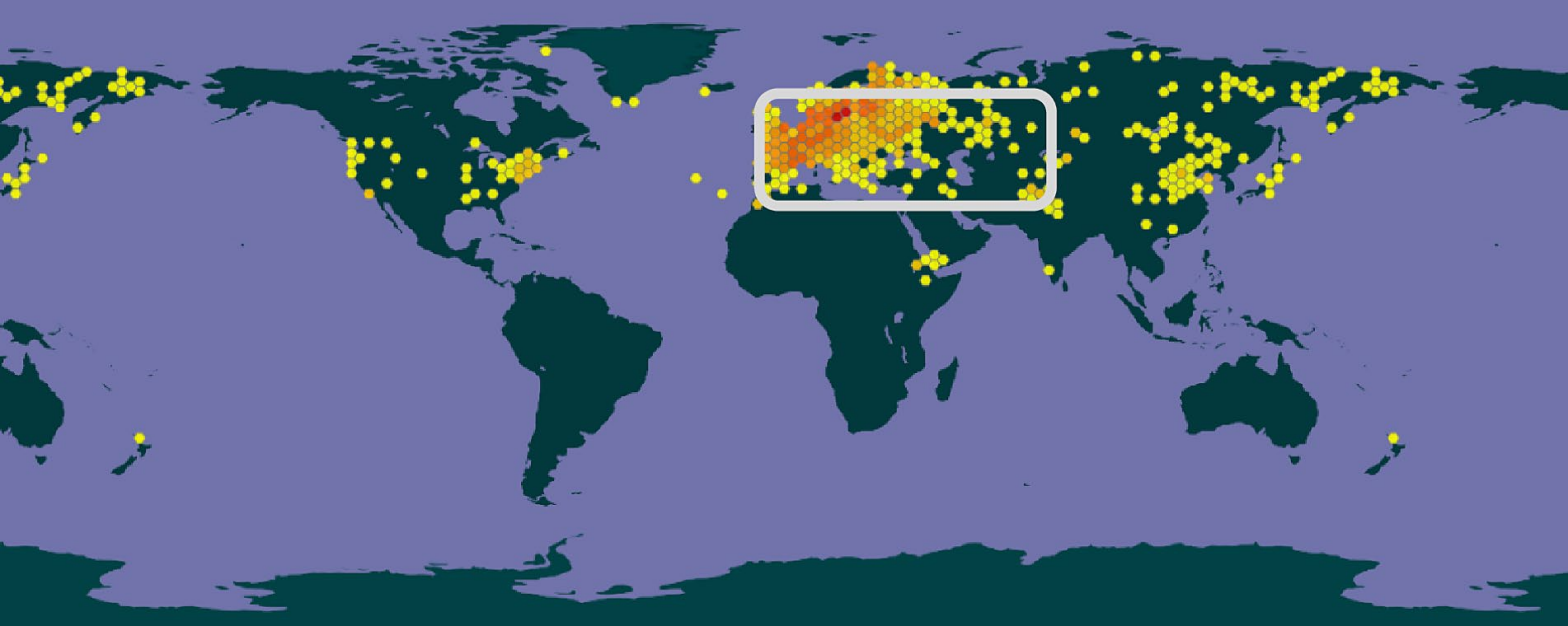
Global map of distribution of *Thymus serpyllum* (19). The area highlighted is the approximate native range of the species and it is now cultivated in a wide region in the Holarctic.

*Thymus serpyllum* and closely related taxa originated in the wider European, North African and Asian (Palearctic) region and have been introduced and distributed globally in regions with a suitable climate. It is cultivated in many world regions for its aromatic essential oil or the herbal part used in food or as medicine in the pharmaceutical industry. Some publications even indicate a broader distribution than shown on the map. On the other hand, some dots represent only a local occurrence in a botanical garden or alike (Figure 3) (19).

#### 3.2.2 Cultivation and propagation

Wild thyme, a hardy (up to −15°*C. minimum* temperature) evergreen shrub, grows up to 0.1 m–0.3 m at a medium rate, flowering from July to August, with the seeds ripening from August to September. The seeds are used for reproduction mostly in late spring after the frost period, early or during summer. Germination is possible using surface sow or barely covered seeds. *T. serpyllum* is a hermaphrodite pollinated by insects. The plant prefers light, well-drained, relatively dry, generally sandy soil resulting in sand thyme as one common name. It tolerates a wide pH range from mildly acid and neutral to basic or mildly alkaline and likes calcareous soil but not so much salty conditions, although it can grow on sand dunes, it can withstand strong winds and droughts and prefers sunny exposures (20). The cultivation techniques are similar to other, more erect, thyme species, like common thyme, an exception of harvesting needs to be mentioned. Wild thyme is a creeping species that requires a more complex harvesting method (21). Chauhan et al. (22) describe a field experiment focusing on the effect of four different harvesting times (115 to 175 days after planting) on growth, yield and quality, especially of essential oil of wild thyme in Uttarakhand, India. During the four harvests, about 9 L/ha, each oil from dried aerial parts with contents between 0.5–0.7% were obtained (22). Heeger (23) quoted the Roman agronomist Palladius’ Treatise on Agriculture (*Opus agriculturae*) in the fifth century, whereas he only describes its planting as an Annex to his *T. vulgaris* chapter.

### 3.3 Economic botany and uses

The popularity of Mediterranean cuisine and the perceived health benefits of thyme are contributing to the growing demand for dried thyme in the European market. European production of dried thyme is not self-sufficient. The leading EU producers are Poland, followed by Spain and France, but large volumes are imported from non-European countries (24). Europe is the largest dried thyme importer in the world, accounting for a 50% share of the world’s total imports. Germany is the largest dried thyme importer in Europe, followed by Spain, Belgium, the United Kingdom, France, and the Netherlands. Around one-third of European dried thyme imports come from North Africa (Morocco and Egypt), Turkey, the Middle East (Israel and Syria), and the Balkans (Albania) (24). A significant share of imported dried thyme is used as a medical herb for the preparation of herbal teas and infusions. Thyme is also used as an ingredient in spice mixes for the food industry, including the potato and sausage industries. The increasing interest in international ethnic cuisines, combined with buyers’ needs for stable and sustainable sourcing, is the leading driving force behind the growing interest in dried thyme in Europe in fair-trade and organic-certified quality (24).

#### 3.3.1 Traditional culinary use

*Thymus vulgaris* L. and *T. serpyllum* L. grow in the same areas (Europe and North Africa) and they have been used since ancient times as seasoning. Although several hundred species and cultivars are grown, among others, the best known are *T. vulgaris* and *T. serpyllum* (25). In East Europe, wild thyme is used in traditional dishes, for instance, in Moldova. Here, wild thyme is an important ingredient to prepare sour borscht (borș) with health-promoting qualities, obtained by fermenting wheat bran. This sour liquid food preparation is rather unknown to the Western but very popular in Eastern countries, e.g., the post-Soviet world. Wild thyme is inevitable for the traditional Moldovan soup called zeama. Also, *T. serpyllum* is used to flavor iron wine, oil or vinegar, whereas the herb with inflorescences, stems, and leaves are used to make tea (26). Wild thyme-flavored spirits are available as well, like the French digestive “Serpolette 25.6% Vol.,” a wild thyme liqueur produced by a company called MAISON 16.

#### 3.3.2 Functional foods and dietary supplements

*Thymus serpyllum* is gaining significant traction in the functional food and dietary supplement markets. This trend is in line with the growing consumer preference for natural and herbal remedies. While *T. serpyllum* is best known for its traditional use in managing and treating respiratory conditions, its incorporation into various product categories has expanded its reach. *T. serpyllum* is often found as a key ingredient in medicinal confectionery products, including throat lozenges and pastilles. These products often target cold symptoms, cough suppression, and throat soothing. Well-known manufacturers have capitalized on this trend. *T. serpyllum* is also found in dietary supplements formulated to support respiratory health. These supplements typically contain a blend of herbs associated with respiratory health. Beyond its medicinal uses, *T. serpyllum* is occasionally included in functional foods and beverages for its aromatic properties, contributing to flavor profiles while offering potential health benefits.

*Thymus serpyllum* is rarely found as a mono-ingredient dietary supplement. Instead, it is often combined with a blend of complementary herbs traditionally associated with cold symptoms and respiratory health. However, an exception to this trend is a liquid product in the USA, which offers *T. serpyllum* as a standalone herbal supplement with no specific indications, serving a niche market segment.

**Figure 4:**
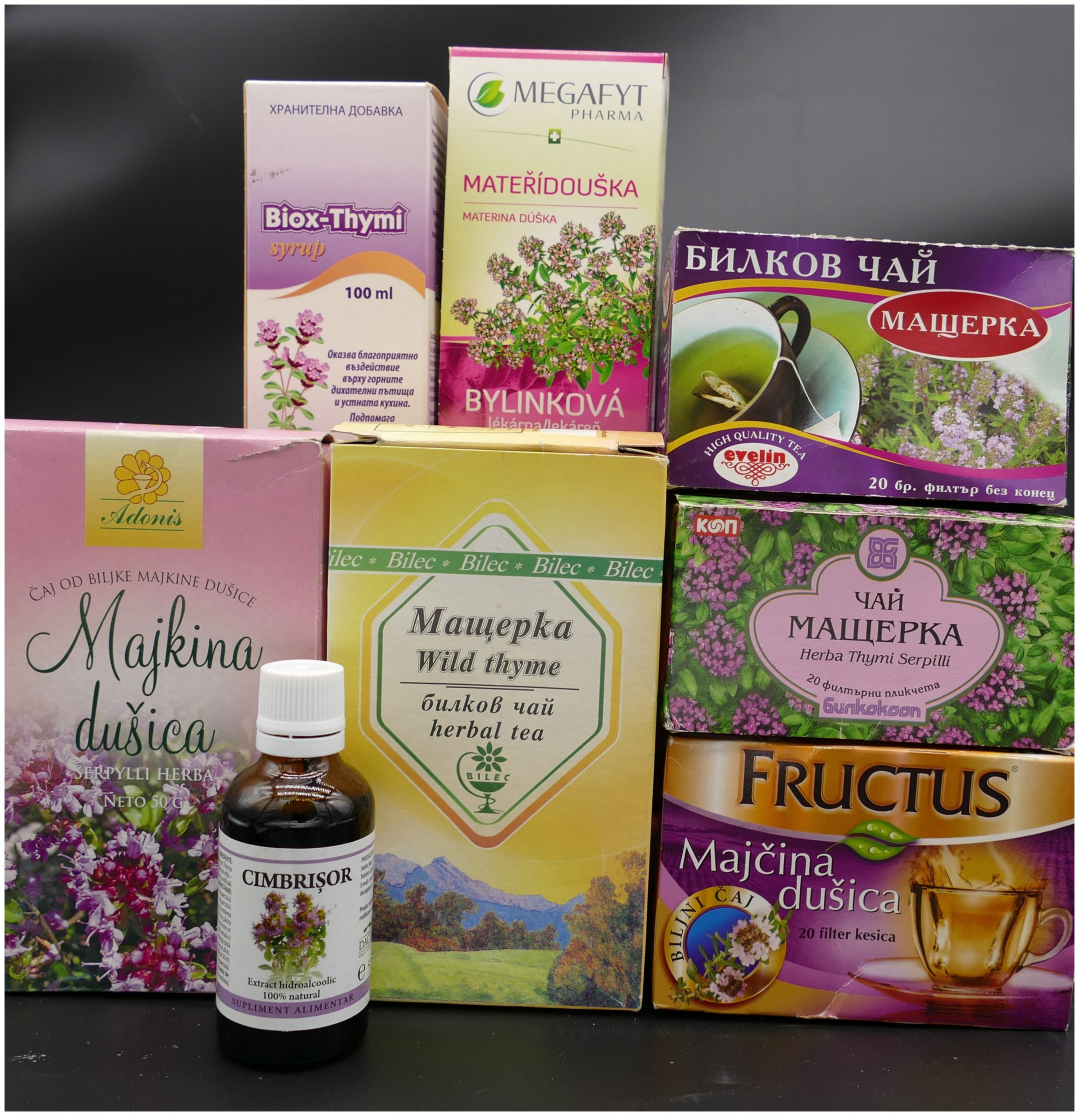
Selection of Central and East European *Thymus serpyllum* (including combination) products (Photo Bjoern Feistel, 2023).

The products are produced mainly in EU countries like Germany, Switzerland, Italy, and the USA. In total, 57 products were found in the Mintel Database. Herbs grown in the Mediterranean climate, including wild thyme, are part of the Mediterranean diet. Several studies suggest that a Mediterranean diet could lead to lowering the risk of cardiovascular diseases, neurodegenerative diseases, diabetes, and early death. Currently, industries around the world are following this trend, and they are offering dietary supplements with digestive & detoxifying treatments, including wild thyme as an ingredient (Figure 4).

#### 3.3.3 Traditional medicinal use

Serpylli herba, a well-documented and widely utilized herbal remedy, has enjoyed historical usage in the management of mild gastrointestinal and dyspeptic conditions. While there is currently no official monograph from the HMPC (Herbal Medicinal Products Committee), or as Hagers Enzyklopädie der Arzneistoffe und Drogen (27), the British Herbal Compendium (28), Herbal drugs (29) and Kooperation Phytopharmaka (30), provide information on wild thyme, including recommended dosage and indications. A Commission E monograph (1987; 1990) (31), specifically addresses wild thyme’s application in treating upper respiratory tract catarrhs. The extensive documentation supports the safe use of wild thyme, with no known contraindications, adverse effects, or interactions with other medications. Furthermore, an ESCOP (European Scientific Cooperative on Phytotherapy) monograph (32) on Serpylli herba documents the safe oral utilization of wild thyme, confirming the absence of interactions, undesirable effects, overdosing, and contraindications. Quality monographs found in several pharmacopoeias also highlight the widespread use of *T. serpyllum*, including traditional usage. These pharmacopoeias include Farmacopoea Official Española (33), DAB (Deutsches Arzneibuch) (34), Farmacopeia Portuguesa (35), Farmacopea Polska (35), Pharmacopoeia Hungarica (36), British Herbal Pharmacopoeia (37), Pharmacopée Française (38), and Pharmacopoeia European (39). In various European countries, finished medicinal products in the form of teas are available in the market. Dosage recommendations vary but can extend up to 9 grams of herbal drug equivalents per day. Additionally, products using extract preparations, either in aqueous or hydroethanolic form, presented in syrups, have also been identified. Wild thyme can be used in the preparation of herbal baths and herbal pillows (1, 40).

#### 3.3.4 Cosmetics and skin care

Thyme oil is commonly incorporated in a variety of cosmetic products, including deodorants, due to its ability to neutralize odors and its antimicrobial characteristics (1, 41, 42). In soap fragrances, its robust and refreshing qualities add a touch of medicinal-like aroma, which is often preferred in specific soap or detergent types. Thyme oil is effective in concealing strong, tar-like smells (43).

When used sparingly in lotions, perfumes, or colognes, thyme oil contributes to a fuller, subtly sweet scent. Consequently, it is a component in the formulation of various cosmetic creams, lotions, eau-de-colognes (often blended with lemon and bergamot), and soap-based solutions for sterilizing surgeons’ hands (44). These products are beneficial in treating acne and other skin issues. Both thyme essential oil and thymol find applications in the production of toothpaste and mouth rinses (45, 46). A 10 percent peroxidised thyme oil solution in soap effectively eliminates oral microbial flora within three minutes (1, 40). Wild thyme is found as an extract in the cosmetic industry. It is recommended for sensitive skin, against itching, and for decongesting the scalp hair as an antiseptic and stimulant of the local blood flow (WILD THYME Liquid B, ICHIMARU PHARCOS CO., LTD. INCI Name: *T. serpyllum* Extract). The beneficial action on the microcirculation is associated with claims related to tired legs relief by protecting the micro capillaries from diverse deterioration and, in this way, facilitates the return of blood flow by maintaining vascular integrity (Tired Legs Complex, Greentech, INCI Name: Aqua (and) Propylene Glycol (and) *T. serpyllum* Extract (and) *Ruscus aculeatus* L. Root Extract). Another product is based on the traditional therapeutic use of wild thyme for wound treatment, general metabolism support and as a ‘fountain of youth’. The plant has a firming effect and is said to help with inflammatory changes in the skin. In this cosmetic product, wild thyme is in combination with lady’s mantle (Achillea spp.) and jujube (*Ziziphus jujuba* MILL.), which provides anti-aging, astringent and moisturizing effects for skin care preparations. Another application in the cosmetic industry for wild thyme is as a whitening agent. Wild Thyme prevents pigmentation by inhibiting the transport of melanin to epidermal cells. Wild thyme can also improve the appearance, texture and fragrance of cosmetic products (47).

#### 3.3.5 Technological application and other uses

In addition to culinary and pharmaceutical uses of *T. serpyllum* preparations, there are several others, such as food preservation, especially for meat, milk, fish and their products or dishes (48) due to the antioxidant action or as an insect repellent or pesticide (49). The latter rely mainly on essential oils with main constituents like thymol or carvacrol (50) but sometimes also non-volatile extracts based on the rather high content of rosmarinic acid or di- and triterpenoids (51).

### 3.4 Regulatory aspects worldwide

The medicinal properties and the traditional use of *T. serpyllum* are recognized in several countries. In 2014, the European Scientific Cooperative on Phytotherapy (ESCOP) published a monograph described *T. serpyllum* for the treatment of upper respiratory tract catarrh as a bath additive to support acute or chronic diseases of the respiratory tract (32). These uses are based on evidence from long-term human use. In 1987 (revised 1990), the German Commission E published a monograph on the cut herb for infusions and other preparations for internal use and for the treatment of catarrh of the upper respiratory tract Commission E monograph (1987; 1990) (31).

Although there is a long tradition of medicinal use in Europe, the Committee on Herbal Medicinal Products (HMPC) of the European Medicines Agency has not established a harmonized monograph for therapeutic uses and conditions of safety, neither for well-established nor for traditional uses (52). However, there is a quality monograph in the European Pharmacopoeia describing the whole or cut, dried, flowering aerial parts of *T. serpyllum* L. with a requirement for essential oil of a minimum of at least 3.0 mL/kg in the dried drug (53).

In addition to its medicinal uses, *T. serpyllum* is used as food, as a culinary spice, or as tea, and also as a food supplement. *T. serpyllum* is included in several positive lists in European countries for the use in food supplements, such as Belgium (54), Croatia (55), France (56), Germany (57) and Italy, where health claims are established for digestive function, regulation of gastrointestinal motility and gas elimination, antioxidant, the well-being of the nose and throat (58). According to the EU Regulation 1924/2006, nutrition and health claims can be made on foods once they have been approved by the European Food Safety Authority (EFSA). However, more than 2000 applications for health claims on botanicals have not been evaluated by EFSA due to concerns raised by several EU member states and industry stakeholders about the different regulatory regimes for health claims in food and food supplements on the one hand and traditional herbal medicinal products on the other. However, these so-called “on-hold claims” can still be used to inform the consumer. For *T. serpyllum*, there are “on-hold claims” for respiratory (soothing for mouth and throat; ID 3716 and 4,166) and gastrointestinal health (improvement of digestive function; ID 4491) for being used in supplements. The combination of *T. serpyllum* with other plants is also used for respiratory health claims (ID 4607 and 4,615) (59). On the other hand, the benefits for the urinary system were considered inappropriate as they are general and do not refer to a specific health claim as required by Regulation (EC) No 1924/2006 (60). In Asian countries, *T. serpyllum* is also recognized as a food ingredient. Buds and leaves of *T. serpyllum* are recognized as *Spices and Condiments* in China (61). In addition, aqueous extracts of edible plants may be considered as food ingredients for the *Botanical Beverages* (62). In South Korea, *T. serpyllum* is listed in Annex I, “*Ingredients Approved for Use in Food*” in Food Code (63), which also states that food may be extracted using only water, alcohol, or a mixture of water and alcohol, and carbon dioxide. In Japan, this species is listed on the *List of ingredient essences (raw materials) that are not considered pharmaceuticals unless they are labeled as having medicinal effects* (64), and its water and ethanolic extracts can still be considered food or food additives. In India, the species is listed in the regulations of the Food Safety and Standards (FSSAI) (65). In the USA, the species is listed in Herbs of Commerce (66), and is recognized as safe for use as a spice and other natural seasonings and flavorings. Its aqueous and ethanolic extracts could commonly be available as a dietary supplement. In addition, *T. serpyllum* and it extracts, and based on the Food and Drug Administration (FDA) (67), and the essential oil is recognized as safe (GRAS) (68).

### 3.5 Patents and intellectual property rights (IPR)

Our search strategy resulted in a list of 15 patents. However, many of these documents show the main term “*Thymus serpyllum*” only as a botanical drug within a long list of plant species (see Supplementary Table S1).

After a successful patent examination procedure, only four of the 15 patent applications were finally granted as patents (indicated by the code “B” at the end of the patent number): RO98315B1, RO98319B1, US11179430B2, CN106344648B. The majority of the patent applications still show *T. serpyllum* only in a long plant list citation but without their own specific data. For example, only one patent is highly relevant because “*Thymus serpyllum*” (or Mother of Thyme) is mentioned in the title, abstract, claims, and the main text body of the document (Pat#6: US11179430B2 = WO2013182709A1), which deals only with *T. serpyllum* extracts, their preparation, and specific use/application (Table 1).

### 3.6 Phytochemistry

#### 3.6.1 Nutritional value

The nutritional composition of *T. serpyllum* herba varies between literature sources and studies, probably due to different varieties, genetic, environmental, ecology and harvest conditions of the plant, as well as the analytical methodology (69) reported that *T. serpyllum herba* from India on dry weight bases contains protein (21.4 g/100 g), fat (5.5 g/100 g), carbohydrates (11.9 g/100 g), starch (6.5 g/100 g) and amino acids (2 g/100 g). The dietary fiber composition of wild thyme herba includes neutral detergent fiber (NDF) (15.1/100 g), acid detergent fiber (ADF) (10.6 g/100 g), lignin (3.6 g/100 g), hemicellulose (4.2 g/100 g) and cellulose (7.1 g/100 g). Vitamins (mg/100 g) are for Vitamin A 4.9, Vitamin C 43.8 and Vitamin E 13.7. Other bioactive metabolites are (Poly)Phenols (86.6 mg/g), Flavonoids (51.9 mg/g), and Phytosterols (45.3 mg/g). Some of the latter contribute to the antioxidant profile of *T. serpyllum*, measured/determined as DPPH (48.58 μg/mL) and FRAP (105 mg/g), which provides analytical-chemical guidance only. The ash content was found to be (2.7 g/100 g) and comprised of the following macro, micro minerals and non-metal elements quantified by e. g., X-Ray Fluorescence (XRF) analysis of the plant powder and other methods, like AAS, AES (Table 2). Some studies indicate that *T. serpyllum* can accumulate trace elements and heavy metals, like lead (Pb), if the soil is contaminated (69–72) (Table 2).

**Table 2:** Mineral and elemental content of *Thymus serpyllum* (69–72).

Minerals	Trace elements	Non-metals/elements	Heavy metals
K 2100*/5,040****	Al 280*/6700****	Si 610*/29100****	Pb 12.0–17.9***
Ca 1850*/8,715****	Fe 160*/142**/207–879***/2,520****	P 200*	Ba 200****
Mg 430*/2,150****	Mn 9.6*/14.7**/132****	S 170*	Sr 86****
Na 20*/2,140****	Cu 9.6*/10.4**/8–16***	Cl 120*	
	Zn 3.8*/15.8**/42–117***		
	Ni 0.8*/ND**/10.8		
	Co 0.4*/1.0****		
	Cr 3.3*		

Mineral and elemental composition in references: *Shahar et al. (69) mg/100 g, **Abu-Darwish (70) ppm, ***Figas et al. (71), ****Vasil'eva et al. (72) mg/kg; Elements: K-potassium, Ca-calcium, Mg-magnesium, P-phosphorus, S-sulphur, Al-aluminium, Na-sodium, Cl-chlorine, Si-silicon, Fe-iron, Zn-zinc, Mn-manganese, Cu-copper, Ni-nickel, Co-cobalt, Cr-chromium, Pb-lead, Ba-barium, Sr-strontium.

#### 3.6.2 Bioactive metabolites

Wild thyme, as an aromatic member of the mint family, contains up to 1% essential oil, representing only a small fraction of the metabolites elucidated to date. Still, the essential oil’s composition and bioactivity have been studied much more extensively than the non-volatiles. An increasing number of metabolites have been elucidated over the last decades. For example, in the early 1990s, Duke (73) listed in his “Handbook of phytochemical constituents of GRAS herbs” 47 metabolites, of which 36 were essential oil compounds and 11 were non-volatile ones. On the other hand, a recent publication by Yang et al. (74) describes 410 metabolites of *T. serpyllum*, of which about 200 are flavonoids, followed by more than 120 phenolic acids. The detection of this large quantity of phytochemicals was possible by applying modern analytical instrumentation such as UPLC-ESI-Q TRAP-MS/MS coupling joint with a suitable data interpretation which also the most recent approach for species authentications and differentiation from other thymus species. Several similar publications failed to do so but reported wrong plant constituents, like silicon derivates and others, which obviously contaminants or artifacts. Many other publications focus more on essential oils and their compounds, although essential oils make up only about five permille of the dry raw material.

##### 3.6.2.1 Essential oils of *Thymus serpyllum*

ESCOP (32) states the essential oil content of *T. serpyllum* with 0.1–0.6%, thus the single constituents within this group are minor ones, but due to the ease of separation by water steam distillation, they were characterized early in history. As mentioned above, about 35 volatile compounds, mainly terpenoids, are described, although additional new essential oil metabolites are occasionally reported due to advancements in analytical methods and techniques. Some authors analyzed the variability of the species *T. serpyllum* in one country (75), others even describe several chemotypes within a certain region (76). Steflitsch et al. (77) summarized about 15 main phytochemicals with their percental ranges, as shown in Table 3.

**Table 3:** Essential oils content of *Thymus serpyllum.*

Essential oil metabolites	Chemical class	% [GC area]	References
	Monoterpene phenols	Σ 9.0–82	(32, 77)
Carvacrol		2.1–80	(77, 78)
Thymol		1.0–16	(77, 78)
	Monoterpene alcohols		(77)
Linalool		0.8–28	(77, 78)
Terpinen-4-ol		0.4–3.7	(77, 78)
Borneol		≤ 4.1	(77, 78)
α-Terpineol		≤ 17	(77, 78)
Geraniol		≤ 12	(77, 78)
Geranyl acetate	Monoterpene ester	≤ 6.8	(77, 78)
	Monoterpenes		(77)
β-Myrcene		0.3-5.4	(77, 78)
γ-Terpinene		2.7-17	(77, 78)
*p*-Cymen		3.6-28	(32, 77)
1,8-Cineole	Monoterpene oxides	≤ 2.0	(77, 78)
Camphor	Monoterpene ketone	≤ 2.0	(32, 77)
	Monoterpene phenol ethers		(32, 77)
Carvacrol methyl ether		≤ 1.2	(77, 78)
Thymol methyl ether		≤ 2.3	(77, 78)
β-Caryophyllene	Sesquiterpene	0.1-4.7	(77, 78)

##### 3.6.2.2 Non-volatile metabolites of *Thymus serpyllum*

Depending on the region/origin, climate, time of harvest, extraction methods and solvents used, the following non-volatile plant constituents (e.g., Polyphenols, flavonoids, phenolic acids, and fatty acids) have been found and are summarized in Table 4.

**Table 4:** Non-volatile content of *Thymus serpyllum.*

Polyphenols/Flavonoids/Phenolic acids + Fatty acids	References
Apigenin	(80–84)
Apigenin-7-O-β-D-glucoside	(1)
Apigenin-4′-O-β-D-p-cumaroyl-glucoside	(1, 85)
Caffeic acid	(1, 81, 86, 87)
(+)-Catechin	(81, 87, 88)
Chlorogenic acid	(81, 88)
p-Coumaric acid	(87)
Coumaric acid hexoside isomer-1+2+3	(87)
3-p-Coumaroylquinic acid	(87)
4-p-Coumaroylquinic acid	(87)
cis-Coutaric acid	(87)
trans-Coutaric acid	(87)
Dihydroxycoumarin	(87, 88)
Diosmetin-7-O-β-D glucuronide	(1)
Ellagic acid	(87)
Eriocitrin	(80, 89)
Eriodictyol	(87, 88)
(−)-Epicatechin	(81, 87, 88)
Ferulic acid	(81, 88)
Gallic acid	(87, 90)
Isorhamnetin-3-O-galactoside	(87, 88)
Kaempferol	(81, 87, 88)
Kaempferol-3-rutinoside	(87, 88)
Kaempferol-3-galactoside	(88)
Kaempferol-3-glucoside	(87, 88)
Lithospermic acid	(89)
Luteolin	(80, 82, 84, 88, 89)
Luteolin-galactoarabinoside	(1)
Luteolin-7-O-β-D-glucoside	(78, 81)
Luteolin-7-O-β-D-diglucoside	(1)
Luteolin-7-O-glucuronide	(89)
Luteolin-7-O-rutinoside	(89)
Monogalloyl-glucose	(87, 88)
Myricetin	(90)
Naringenin	(81, 87)
Naringenin-7-O-glucoside	(87, 88)
1-Octen-3-ol	(78)
3-Octanol	(78)
Oleanoic acid	(78, 86)
Protocatechuic acid	(87)
Quercetin	(80, 81, 87)
Quercetin hexoside isomer-1+2	(87, 88)
Quercetin-3-O-glucoside	(87, 88)	Polyphenols/Flavonoids/Phenolic acids + Fatty acids	References
Quercetin-3-O-rutinoside	(87, 88)
Quercetin glucuronide	(87, 88)
Quercetin-3-O-galactoside	(87, 88)
Quercetin pentoside isomer-1+2	(87)
Rosmarinic acid	(80, 81, 88, 89)
Rutin	(81)
Serpyllin (5-Hydroxy-2′,3′,4′,7,8-pentamethoxyflavone)	(78, 80, 86)
Scutellarein	(1, 78, 82)
Scutellarein-glucosyl glucuronide	(1)
Scutellarein-7-O-β-D-glucosyl (1–4) α-L-rhamnoside	(1, 85)
Scutellarein heteroside	(78, 86)
Tannins	(78)
Undecanoic acid	(78, 86)
Ursolic acid	(78, 86)
Vanillic acid	(87)

**Figure 5:**
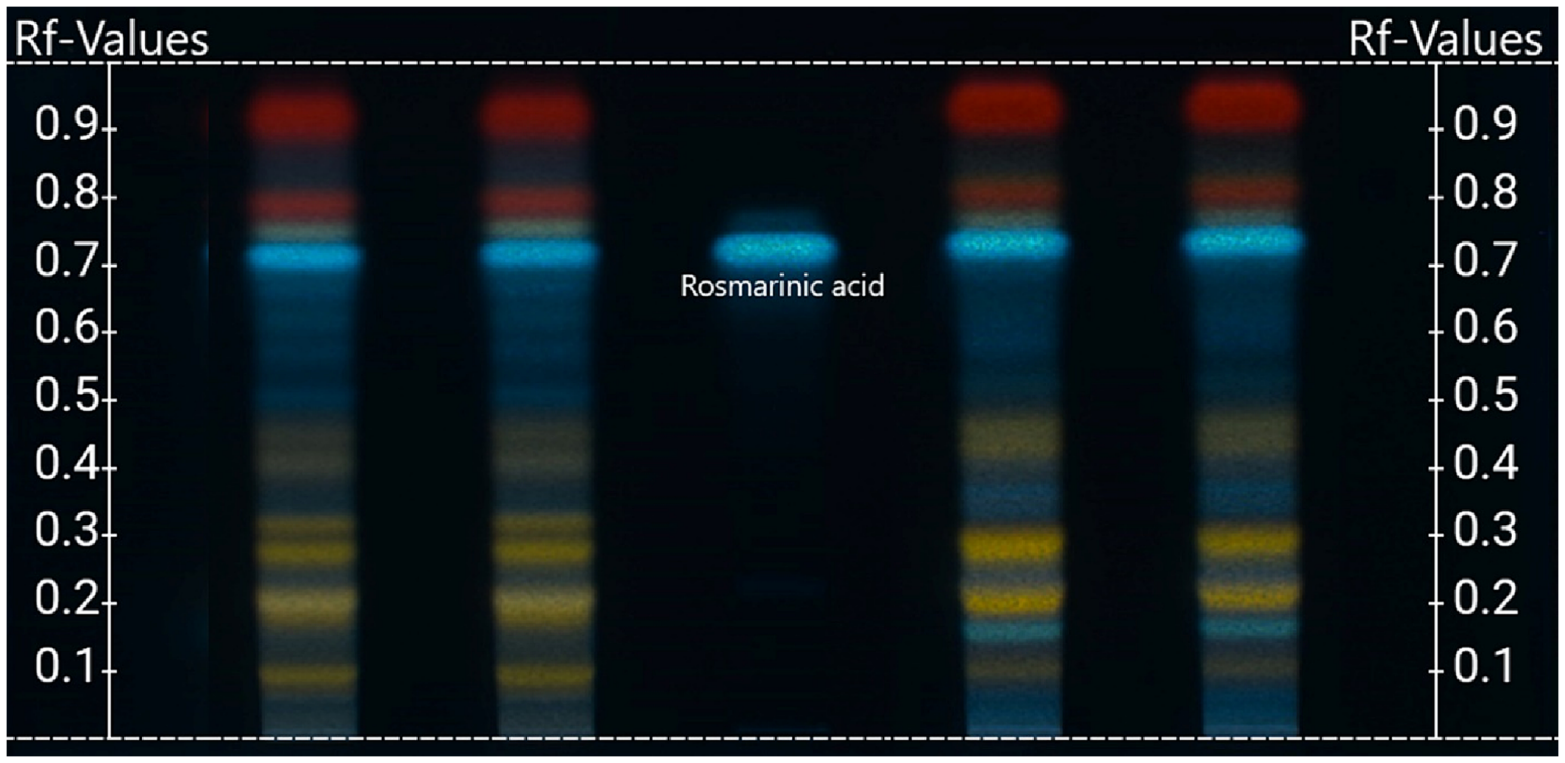
HPTLC image of differentiation of *Thymus serpyllum* from *Thymus vulgaris/zygis* (79), used in the Standard Operation Procedure (SOP) for methanolic extracts including *T. serpyllum* and *T. vulgaris* by Finzelberg, Germany (Finzelberg-SOP-No. 5551). *T. serpyllum* (two lots in the 2 left lanes) and *T. vulgaris/zygis* (two lots in the 2 right lanes) plus reference substance rosmarinic acid in the middle lane; after derivatization and heating in UV-light at 365 nm.

High Performance Thin Layer Chromatography (HPTLC) analysis was applied for the separation of phytochemicals of *T. serpyllum* (methanolic extract) in comparison to *T. vulgaris/zygis* according to the method as described by Meier (79). This methodology could be used to authenticate *T. serpyllum* raw materials from the other thyme species. In this method, as shown, the two thyme species can be differentiated by their specific pattern of HPTLC bands after development and visualization, as seen in Figure 5.

In some cases, particularly for non-regulated commercial products, the presence of other Thymus species instead of *T. serpyllum* cannot be complete, considering its presence in spontaneous flora of various countries and its citation as rare or uncertain. It is impossible to assess this in any review, and also calls for better documentation of what is being studied. In addition, the difference in pharmacopoeial regulations regarding Serpylli herba and its origins may contribute to this confusion, including several national pharmacopoeias recommending the substitution with the aerial parts of other spontaneous Thymus species. On the other hand, official pharmacopoeias mainly regulate the use of pharmaceuticals thus the correct species labeling of the pharmaceutical products is required by law, with not strictly applied to non-regulated pharmaceutical products, like food or dietary supplements, hence, a confusion of *T. serpyllum* with other thymus species cannot be excluded, especially if they are labeled only thyme or wild thyme, because a number of different plants can be meant. At the same time there is also no control over the quality level of starting material used.

### 3.7 Biological and pharmacological activities

#### 3.7.1 *In-vitro* studies

##### 3.7.1.1 Antimicrobial activity

Many essential oils exhibit antibacterial and antiviral activities in various *in-vitro* models. These oils have been tested against pathogenic and non-pathogenic organisms. Several studies assessed the antimicrobial activity of *T. serpyllum* essential oils individually (76, 91–96) or in a mixture design enriched with other components including with other essential oils (92, 97, 98) using, for example, different bioassays, agar well/disk diffusion, broth dilution methods including determining the Minimum Inhibitory Concentration (MIC) and Minimum Bactericidal Concentration (MBC). In these studies, the tested concentrations varied between 5–15 μL showed activity against a broad spectrum of microorganisms with *T. serpyllum* essential oils. For example, the antibacterial activity with MIC (0.0625; 0.125% v/v) against *Escherichia coli* and *Staphylococcus aureus* (97), MIC (0.012–0.2%) and MBC (0.012–0.2%) (99) were studied. The strain of *Pseudomonas aeruginosa* showed slightly more sensitivity to *T. vulgaris* and *T. serpyllum* essential oils (MIC 0.5 mg/mL) (100). The antimicrobial activity against a broad spectrum of microorganisms was attributed to the high content of aromatic monoterpenoids thymol (95). In a different study, *T. serpyllum* essential oil in the range of concentration showed MIC/MBC (3.125–6.25/12.5 μL/mL) levels for gram-negative bacterial strain (102). *T. serpyllum* showed antimicrobial activity against oral pathogens, with the possible use for oral care (103), with the MIC (2.5–5 μg/mL), and MBC (5–10 μg/mL) for bacteria and MIC (1–2 μg/mL), and MFC (2–4 μg/mL) for fungi (93). The plant population of *T. serpyllum*, located in sunny meadow conditions, could have a significant content of the aromatic monoterpenes carvacrol and p-cymene, and has shown to have a wider spectrum of antimicrobial activity (76).

The secondary essential oils (hydrosolic essential oil fraction) of *T. serpyllum* were also found to have stronger antibacterial and antifungal activities, and they were richer in phenolic compounds (92.3%) compared to their content in primary essential oils (water insoluble essential oil fraction after steam-distillation) (42.1%) (104). N-Propyl rosmarinate, an isolate from *T. serpyllum*, showed the most potent antibacterial activity compared to other isolates (105). *T. serpyllum* essential oil in the vapor phase also showed antibacterial activity (33–350 μL/L); however, it was less effective compared to other investigated essential oils in the study (106).

The ethanol and chloroform extract of *T. serpyllum* were investigated in a number of *in-vitro* assays for their antibacterial activities (81, 91, 107–110). An ultrasonic 70% ethanolic extract of *T. serpyllum* in a concentration range of 0.0625 to 20 mg/mL showed bactericidal or bacteriostatic activity against a range of tested bacteria and fungi. However, there is a difference in the strength of action against strains of microorganisms (81), and the ethanolic extract was considered equally effective against gram-positive and gram-negative bacteria compared to clove oil (108). The bactericidal activity of the aqueous ethanolic extract of *T. serpyllum* for gastrointestinal disorders in a concentration range of 7.8 μg/mL – 500 μg/mL showed growth inhibition of *Heliobacter pylori* at 500 μg/mL in all strains (111). However, with such a high concentration needed, therapeutic benefits are unlikely. Different extraction methods (e.g., CES, UAE, and MWE), with impact on the antimicrobial activity of *T. serpyllum* were also investigated (112). *T serpyllum* exhibited the highest inhibition rate of 132.93% against *Escherichia coli*, and 78.40% against *Candida albicans* compared to other investigated medicinal plants (112).

The anti-fungal and anti-Candida activity of *T. sepyllum* has also been tested in several *in-vitro* models. The MIC value of *T. serpyllum* was 0.039 to 0.078% against tested bacteria and fungi, in addition to this, different combinations of essential oils and thymol with antibacterial and antifungal antibiotics were found to increase the efficacy of the antibiotics by 4–130 folds against bacterial and fungal pathogens (94). The mycelium was found to be less affected in the presence of *T. serpyllum* essential oil using Mycelial Growth Inhibition assay; at 0.25 mg/L, the MGI were 99%, while at the 0.5 mg/L of *T. serpyllum* essential oil, the MGI value drops to 22% (113). In contrast (114), reported that *T. serpyllum* did not show potent anticandidal or antifungal activity against tested strains. *T. serpyllum* essential oil samples were tested for their antifungal activity in human and animal skin, the MIC value of the fungi species was in the range of 0.5- > 10 mg/mL and the MFC were in the range of 1.25- > 10 mg/mL. *T. serpyllum* oil showed strong antifungal activity, especially against dermatophyte fungi (115). In a different study, *T. serpyllum* showed antifungal activity against oral isolates of *Candida albicans* and *Candida glabara* with a MIC value of 500–1,000 mg/L (116) (for more details, see Supplementary Table S2).

##### 3.7.1.2 Anti-inflammatory activity

The anti-inflammatory activity of aqueous, ethanolic and methanolic extracts of *T. serpyllum* were tested in a number of assays, assessing the enzymes linked to inflammation, such as hyaluronidase, lipoxygenase (for inflammatory skin diseases) and src tyrosine kinase inhibitions (117, 118). At a concentration 150 μg/mL, the aqueous extract of *T. serpyllum* showed 71.7 ± 4.9% inhibition of the hyaluronidase activity, and it was dose dependant, and the IC50 value was 118.1 ± 7.1 μg/mL (118). In addition, the methanolic extract of *T. serpyllum* has been shown to inhibit the Src tyrosine kinase activity (for more details, see Supplementary Table S2).

##### 3.7.1.3 Other pharmacological activities

The aqueous and methanolic extract of *T. serpyllum* were tested for a number of different pharmacological activities, e.g., chemopreventive activities and as an adjunct therapy for the cancer (119–121). Aqueous tea infusions and essential oils has shown to ameliorate the oxidation susceptibility of low-density lipoproteins (LDL) in a dose-dependent manner as found in a copper-induced LDL oxidation *in-vitro* model (122), antithrombin activity (123), antidiabetic/alpha-glucosidase inhibition activity (124, 125), liver and hepatoprotective activity (126) and anticholinesterase activity (127). The ethanolic and infusion extract of *T. serpyllum* were also looked at for its skin protective (photoprotective) activities, and it was the extract exhibited high values of Sun Protective Factors (SPF) with 38.34 ± 2.29 and 38.82 ± 2.23 for ethanol and infusion extract, respectively. These results suggest a potential use of *T. serpyllum* as a source of bioactive compounds with skin-protective properties (117, 128) (for more details, see Supplementary Table S2).

#### 3.7.2 Pre-clinical (*in-vivo*) studies

##### 3.7.2.1 Cardiovascular disease and metabolic syndrome

Several preclinical studies have been published on cardiovascular diseases, including antidiabetic (129–131), antihyperlipidemic and liver enzyme protective activities (132–134) of the aqueous extract of *T. serpyllum*. Ether and aqueous extract were found to reduce the blood glucose in diabetic rabbits (500 mg/kg b.w. with glibenclamide and acarbose as control), with aqueous extract inhibiting the rise in glucose level in the oral tolerance test (129). The extract showed a synergistic effect with different insulin levels; the HbA1c level was reduced, whereas the hemoglobin level was increased in the 3 months study (129).

Another study by the same research group in the same setting treated diabetic rabbits with a single high dose of 500 mg/kg of the aqueous extract. Blood samples were taken on day 0 and day 30. The extract has been shown to reduce the level of serum cholesterol, triglyceride, LDL, VLDL, alkaline phosphatase and transaminases without affecting the HDL level. The total cholesterol/HDL- ratio was significantly reduced as compared to diabetic control (132). These findings are unlikely to be of therapeutic relevance. This is due to the high dosage tested in this study, for examples, if we compare rabbit dose in mg/kg to human dose in mg/kg. With a rabbit to human conversion factor of 500/3.1, the human equivalent dose would be 161 mg/kg x 70 kg (human body weight) which implies a single dose of 11.3 g, making the study Alamgeer et al. (132) simply implausible (135).

A diabetes model of BALB/c mice fed with a high-fat diet and two streptozotocin (i.p.) injections was employed, and the mice were administered daily with an aqueous extract of *T. serpyllum* (500 and 800 mg/kg/d) for 4 weeks. This treatment was found to be significantly effective in controlling hyperglycemia and improving glucose and insulin tolerance. Within isolated liver tissue, upregulated mRNA levels of the AMPK, IRS1, and GLUT2 gene expression were elucidated. In addition, the cellular morphology of the liver, kidney, and pancreas was restored as revealed by histopathological examination (136). *T. serpyllum* mediated nanoparticles (10 mg/kg) have also shown strong antidiabetic activity on streptozocin-Induced diabetic BALB/c mice models, where it increase the AMPK and IRSI expressions, hence the increase update of glucose in cells (131).

The aqueous extract has also shown membrane-stabilizing properties in Wistar rat models (134). The mixture of commercial lactobacteria and *Lactobacillus helveticus* with water-soluble extract of *T. serpyllum* on a sterile milk basis has also appeared to have protective activity against toxicity and inflammation to the liver (133). The phytobacterial agent (150 mg/kg) seems to improve the microbiological indicators of the intestinal flora and the morphological parameters of the liver of white rats with intestinal dysbacteriosis (induced by the carbon tetrachloride and ampicillin trihydrate) (133).

The aqueous extract was also shown to have antihypertensive activity and decrease vascular resistance in hypertensive and normotensive rats (100 mg/kg b.w. as i.v. bolus in 0.2 saline) (130). This inverse correlation between vascular resistance and plasma heme oxygenease-1 was attributed to endogenous vasodilator carbon monoxide generated by heme oxidation could account for the normalization of blood pressure (130). The antihypertensive effect of the aqueous extract of *T. serpyllum* was elucidated with an injection of 100 mg/kg b.w. as i.v. bolus in 0.2 mL saline — induced a decrease of systolic and diastolic blood pressure and total peripheral resistance in spontaneously hypertensive rats (SHR) without effects on these parameters in normotensive Wistar rats. The results suggest that the aqueous extract may protect against hypertension in the experimental model of essential hypertension, while the cardiac index remained unchanged at the treatment in all experimental rats (137) (for more details, see Supplementary Table S3).

##### 3.7.2.2 Anti-inflammatory activity

The extract of *T. serpyllum* extract was tested in a few *in-vivo* models (138–141). The aqueous extract showed intestinal anti-inflammatory effects in both experimental models of colitis (mimicking inflammatory bowel disease, IBD), as shown histologically as it facilitated tissue recovery of damaged colon and biochemically, as appeared by the improvement of the different inflammatory biomarkers, including myeloperoxidase activity, glutathione content, and leukotriene B4 levels as well as the expression of the inducible proteins iNO2, and COX-2 (138). Trinitrobenzenesulfonic acid models of rat colitis and dextran sodium sulfate model of mouse colitis, where two groups were given the extract at the doses of 100 or 250 mg/kg, and one group was treated with sulphasalazine at the dose of 50 mg/kg. The beneficial effect of *T. serpyllum* was associated with the reduction in the expression of different cytokines, e.g., TNFα, IL-1B, IFNy, IL-16, IL-17, the chemokine MCP-1 and the adhesion molecule ICAM-1. Thus ameliorating the altered immune response associated with the colonic inflammation (138).

The Codelac broncho syrup appears to have anti-inflammatory activity in the acute carrageenan inflammation of the paws in rats (139). The intragastric application of Codelac broncho syrup with *T. serpyllum* (0.17 mL/4 times a day), in comparison with fenspiride appeared to reduce paw volume increment compared to that of the control group starting with 4 h after the initiation of oedema by 42.4% (139).

The anti-inflammatory activity and gut dysbiosis of standardized extract of *T. serpyllum* on 70% native extract (DER native 4–8:1) and 30% dextrin were tested in a high-fat diet-induced obesity mice model (150 mg/kg) (142). The study showed that mice that consumed a high-fat diet exhibited thiobarbituric acid reactive substance (TBARS) values higher than standard diet groups; hence, administration of the extract to the high-fat diet-fed mice reduced TBARS values. Therefore, the daily consumption of the extract to high-fat diet-fed mice reduces the weight gain from day 6 onwards at all doses tested, considering the food intake was similar in all high diet-fed mice groups throughout the experimental period (142). The same group of researchers (143) elucidated anti-inflammatory and visceral analgesic benefits with the same extract mentioned above in a rat model of irritable bowel syndrome (IBS) (143) (for more details, see Supplementary Table S3).

##### 3.7.2.3 Other pharmacological activities

Two earlier studies looked at the aqueous and ethanolic extracts of *T. serpyllum* for their antihormonal activity in male Wistar rat models (144, 145). It was found that the antithyrotropic and antithyroidal activity of a variety of plant extracts, including of *T. serpyllum* was accompanied by an additional prolactin diminution as well as the activity of different plant extracts depended on the extraction procedure, e.g., the extraction of powdered leaves with boiling water or ethanol yielded freeze-dried extracts without thyroid hormone-lowering capacity. The chemical oxidation of a hot-water (100°C) extract by KMn0_4_ served to reintroduce the antihormonal activity (145). However, further studies are needed to define the active components of the extracts utilized in these studies and the mechanism of action for this effect (for more details, see Supplementary Table S3).

#### 3.7.3 Clinical studies

##### 3.7.3.1 Gut, digestive, and liver disorders

A recent human pilot study investigated the effects of an aqueous *T. serpyllum* herbal extract on gut health. In a Randomized Controlled Clinical Trial with 40 overweight subjects (N = 2×20) affected by functional gastrointestinal disorders (FGIDs), referred to as gut-brain interaction disorders, took 600 mg of aqueous *T. serpyllum* herbal extract before breakfast for eight weeks (146). The results of the study indicate that wild thyme has the potential to improve gastrointestinal symptoms and increase stool frequency. This also resulted in an improved quality of life. The stool microbiome of the study group was characterized by a high Firmicutes to Bacteroidetes ratio, which could be positively influenced by the intake of aqueous *T. serpyllum* herbal extract (146).

### 3.8 Safety

#### 3.8.1 Toxicology and interactions

*Thymus serpyllum* and established preparations derived from the aerial parts are safe for use in foods, supplements, and herbal medicines when quality control is assured. No safety concerns have been identified in the literature reviewed, and extracts are generally recognized as safe and, as such, are GRAS-listed by the FDA (147). There are no known restrictions on the duration of use or contraindications for oral administration. No special warnings or precautions for use are required, and no drug interactions have been reported (32). No adverse and/or undesirable effects have been reported except for one potential report of acute kidney injury in a female over 75 years old in 2011, which is available on the VigiAccess-WHO website, and no cases of overdose have been reported. As a valuable culinary herb and spice, many food recipes rely on the addition of *T. serpyllum* to various dishes, soups, salads, marinades and sauces and define a framework for safe use. Based on general knowledge of the hazards of undiluted essential oils, wild thyme may cause irritation to the skin and mucous membranes (1).

As with other botanicals containing essential oils, caution should be exercised with enriched or pure essential oils (for detailed information on the antimicrobial effects of the essential oils, see Supplementary Table S2). Effects on microorganisms are also shown in the pharmacological activity part. Fumigant activity against food crop pests (148) and houseflies (49) is well established. *In vitro*, the cytotoxicity of extracts (e.g., containing rosmarinic and lithospermic acid) in cancer cell lines (149), particularly in human breast cancer (119), and human leukemia cells (120) has been investigated. Different cytotoxic modes of action of polyphenols in extracts and pure phenols (thymol, carvacrol) on different cancer cell lines are known; normal and healthy cells seem to be not negatively affected less or not negatively impacted (3). Extracts exert antigenotoxicity and genoprotective effects, e.g., in human fibroblasts (150).

In general, teas and tea-analogs preparations and related dry extracts are very safe based on their historical use, e.g., the daily intake of *T. serpyllum* is reported to be up to 12 g of herbal tea acc. to BHP 1983/Potter 2002. Even extracts prepared with pure ethanol and tested for toxicity according to the OECD 423 guideline do not show any acute toxicity in mice at doses up to 2000 mg/kg (151). On the other hand, a methanolic extract exerts significant activity in brine shrimp lethality assay and antitumor assay, which is interpreted as a valuable anticancer principle (152). The essential oil of *T. serpyllum* reveals strong anticancer properties, as reported by Nikolic et al. (93).

The essential oil’s composition of Thymus species, including *T. vulgaris*/*zygis* and *T. serpyllum* varies between different chemotypes, although they are considered similar under safety aspects. Mixtures of the constituents, e. g. thymol plus carvacrol appear to behave differently and be less toxic than Thymol alone. Many essential oils of Thyme spp. are GRAS, i.e., not mutagenic, not embryotoxic in mice and show no skin irritation up to 2% in fragrance-sensitive volunteers. These results suggest that thyme oil (Thymol/Carvacrol rich) is less toxic than would be expected of thymol and carvacrol alone and from the data on their individual toxicity data. The Commission E Monographs thyme is considered with two approved (positive) monographs: *Thymi herba* (*Thymus vulgaris*) and Serpylli herba (*T. serpyllum*) (31, 68). There is no data available with regard to interaction risks since there is only a single intervention study.

### 3.9 Scientific applications and translational research

In light of the burgeoning exploration of botanical concoctions and their influence on gastrointestinal wellness and microbiome composition, the compatibility between probiotics and plant extracts has emerged as a pivotal topic of inquiry. Pertinent to this discussion is the case of aqueous wild thyme extracts. The seminal work of Vitas et al. (153) offers compelling evidence in this regard. Their study, focusing on the kombucha fermentation of thyme tea, observed a notable increase in titratable acidity. This phenomenon is indicative of the metabolic vigor of the probiotic strains present in kombucha, thereby underscoring the potential synergistic relationship between such herbal extracts and probiotics.

## 4 Conclusion

Wild thyme (*T. serpyllum* L.), a herb native to Eurasia and North Africa, now cultivated globally, is renowned for its essential oil with thymol or carvacrol as key components. While the essential oil content is less than 1% in dry herbs, the non-volatile phytochemicals, primarily rosmarinic acid, phenolic acids, flavonoids, di- and triterpenoids, significantly contribute to its wider commercial potential, including its nutritional value and preventive health benefits. These compounds have been shown to be effective in preventing, managing and treating several health conditions, making wild thyme a valuable functional food and a component in cosmetic and skin care products.

The chemical composition of the plant, influenced by geographical and climatic factors, underpins its health benefits and traditional use in culinary, nutritional, medicinal, and aromatic applications. Traditionally used in local and traditional medicine, particularly for respiratory and gastrointestinal conditions, wild thyme has been the subject of extensive biochemical research and scientific studies, including *in vitro*, *in vivo*, and some clinical trials. These studies have confirmed specific anti-inflammatory, hepato-protective, and cardiovascular benefits, including its role in managing and treating metabolic syndrome. Of particular significance is a recent human clinical trial that demonstrated wild thyme’s effectiveness in improving gastrointestinal symptoms and overall gut health, a finding that builds upon its previously established preventive and therapeutic health benefits in rodent models for inflammatory bowel diseases and irritable bowel syndromes. With an excellent safety and tolerability profile, wild thyme presents a promising avenue for various therapeutic, preventive and commercial applications.

Despite encouraging evidence, further rigorous controlled intervention studies with well-characterized preparations of wild thyme (7) as well as well-designed experimental protocols and models are necessary to fully understand and harness its therapeutic potential as well as preventive health benefits.

## Data availability statement

The original contributions presented in the study are included in the article/Supplementary material, further inquiries can be directed to the corresponding authors.

## Author contributions

BJ: Conceptualization, Methodology, Writing – original draft, Writing – review & editing. IP: Conceptualization, Methodology, Writing – original draft, Writing – review & editing. BF: Writing – original draft, Writing – review & editing. CS: Methodology, Writing – original draft, Writing – review & editing. AB: Writing – original draft, Writing – review & editing. RS: Writing – original draft, Writing – review & editing. RR-E: Writing – original draft, Writing – review & editing. MH: Conceptualization, Methodology, Writing – original draft, Writing – review & editing.

## In memoriam

We dedicate this paper to Prof. Peter Houghton, formerly Kings College London and a vicar of the Church of England, who passed away on 25.04.24 in Hereford, UK. He will be remembered as a wonderful colleague and great pharmacognosist/ethnopharmacologist.
