# Supplementary material for: Wild thyme (Thymus serpyllum L.): a review of the current evidence of nutritional and preventive health benefits*
Source: Front Nutr. 2024 May 23;11:1380962. doi: 10.3389/fnut.2024.1380962 (PMC11153689; doi:10.3389/fnut.2024.1380962)
Supplement: Supplementary file 1 [file Table_1.docx]

| Table S1 Patent Table 1 (Number of hits for patent search strategies 1 & 2) | | | | | | | | |
| --- | --- | --- | --- | --- | --- | --- | --- | --- |
| **Patent search strategies:** |  | **Patent search strategy 1:**  **IPC A61** patent class (& all subclasses) for 'Medical and Veterinary Science, Hygiene' use - including medicinal teas **AND** the terms: **'thymi' AND 'serpylli'** (includes thyme or thyme or serpyl or serpolet etc.) | |  | **Patent search strategy 2:**  Term: **“thymus serpyllum”** **combined with**:  1. **A61K36/53**: IPC for Lamiaceae  2. **A61K2236/30**: IPC for Extract/ion  3. **A23F3/16**: IPC for Tea extraction & instant / tea extracts; treating, making of | | | |
| **Patent databases =>** |  | **Fampat** | **Remarks** |  | **Google Pats** | **Espacenet** | **USPTO** | **Remarks** |
| **↓ Terms for Patent search strategy 1:** |  |  |  |  |  |  |  |  |
| A61 AND  'thymi' AND 'serpylli' |  | 5 revelant of 71 hits | Mainly pats with T. serpyl. in long plant lists a/o its essential oils in technical formulations; latter excluded, as well as duplicates =>  see Pat# 1, 2, 3, 4, 6 in Pat Tab 2 |  |  |  |  |  |
|  |  |  |  |  |  |  |  |  |
|  |  |  |  |  |  |  |  |  |
| **↓ Terms for Patent search strategy 2:** |  |  |  |  |  |  |  |  |
| thymus serpyllum |  |  |  |  | 4,454 / >100k | 516 | 423 | Mainly pats with T. serpyl. & spp. in long plant lists |
| “Thymus serpyllum” |  |  |  |  | 1,623 | 489 | 408 | Mainly pats with T. serpyl. in long plant lists |
|  |  |  |  |  | More relevant pats of the Lamiacea “T. serpyllum” | | | |
| “Thymus serpyllum”; AND A61K36/53; |  |  |  |  | 75 | 114 | 74 | Ditto. |
|  |  |  |  |  | Thymus serpyllum EXTRACTs | | | |
| “Thymus serpyllum” AND A61K36/53 AND A61K2236/30 |  |  |  |  | 7 relevant of 9 hits =>  see Pat# 5, 6, 10-14 in Pat-Table 2 | 2 => see Pat# 6, 12 | 1 => see Pat# 6 | Extract/ion/s of the Lamiacea T. serpyllum |
|  |  |  |  |  | Thymus serpyllum TEA Extracts | | | |
| “Thymus serpyllum”  A23F3/16  (= Tea extraction; Tea extracts; Treating tea extract; Making instant tea) |  |  |  |  | 4 relevant of 5 hits => see PAT# 7-10 in Pat-Table 2 | 0 | 1 => see Pat# 10 | TEA extract/ion/s of the T. serpyllum |
| “Thymus serpyllum” A61K36/53 A23F3/16 |  |  |  |  | 1 => Pat# 7 | 0 | 0 | TEA extract/ion/s of the Lamiacea T. serpyllum |
| Abbreviations: pat/s, patent/s; Ts, Thymus serpyllum; IPC, International Patent Classification; CPC, Cooperative Patent Classification; EO/s, Essential Oil/s; AB, Abstract, GglPat, Google Patent. | | | | | | | | |
