# Supplementary material for: Wild thyme (Thymus serpyllum L.): a review of the current evidence of nutritional and preventive health benefits*
Source: Front Nutr. 2024 May 23;11:1380962. doi: 10.3389/fnut.2024.1380962 (PMC11153689; doi:10.3389/fnut.2024.1380962)
Supplement: Supplementary file 2 [file Table_2.docx]

| **Table S2** Summary of the reported In-vitro studies for Thymus sepyllum essential oils and extracts (n=39) | | | | | | | |
| --- | --- | --- | --- | --- | --- | --- | --- |
| **Activities investigated** | **Extract** | **Model** | **Dose range tested** | **Control used** | **Duration of study** | **Outcome** | **References** |
| Chemoprevention and adjuvant therapy of cancer | Aqueous extract of T. serpyllum | In vitro  Human Jurkat T leukemia cell line | 10, 50, and 100μg/ml | Untreated Jurkat T cells were applied as a reference negative control. Jurkat T cells treated exclusively with  staurosporine were used as a reference positive control | 20 hrs | *T. serpyllum* extract showed a stimulatory effect on the cells’ apoptosis. The incubation of Jurkat cells with 50 and 100 μg/ml concentrations raised the percentage of the cells undergoing apoptosis, compared to the control cells and the cells exposed to the lowest concentration e.g., 10 μg/ml. This effect was the most evident at the highest concentration (2.9-fold increase in early apoptosis compared to negative control, and when total apoptosis (early and late) was considered (3.4-fold increase in comparison with negative control). *T. serpyllum* enhanced the proapoptotic effect of staurosporine at its highest concentrations of 100 μg/ml. Preincubation with the extract increased the number of apoptotic cells by over 17%. | (Berdowska et al., 2022) |
| Antioxidant and anti-inflammatory  (Inflammatory skin diseases/wound healing) | Aqueous and ethanolic extract of L serpyllum | In vitro  Anti-inflammatory effect on the activity of enzymes, such as hyaluronidase (HYAL) and lipoxygenase (LOX), was also performed. | 50, 150, 300, and 500 µg/mL (antiinflammatory) (Anti-hyaluronidase activity) | Castalagin at a concentration of 10 µg/mL (10.7 µmol/L) (Anti-hyaluronidase activity was measured using the turbidimetric method  As apositive control, allopurinolat a concentration of 25  g/mL (183.7  mol/L) |  | *T. serpyllum* aqueous extract at a concentration of 150 g/mL showed 71.7±4.9% inhibition of hyaluronidase activity. The inhibition was dose-dependent, and the IC50 value was 118.1±7.1 g/mL. The activity of the tested extracts was much lower than in the case of castalagin at a  concentration of 10 g/mL (10.7 mol/L, 98-0.34%). | (Mainka et al., 2021) |
| Anti-inflammatory activity | Methanolic extract of T. serpyllum | In Vitro  -Src kinase inhibition  -IL‑6 inhibition in mice splenocytes | - 1000–0.02 μg/mL  - 200–0.8 μg/mL | -Staurosporine (460-0.01 ng/mL)  - Azithromycin  (100-0.39 μM) |  | *Thymus* extracts inhibited Src tyrosine kinase activity and reduced the production of the proinflammatory cytokine interleukin-6 in Balb/C mouse splenocytes. | (Kindl et al., 2019) |
| Cell Proliferation, apoptosis and epigenetic events in human breast cancer cells | Methanolic extract of *T. serpyllum* | In Vitro  The effects of T. serpyllum on apoptosis and epigenetic events in breast cancer cells. XTT cell viability assay was used to determine cytotoxicity. DNA fragmentation and caspase 3/7 activity assays were used in the assessment of apoptosis. DNA methyltransferase (DNMT) and histone deacetylase (HDAC) activities were evaluated by ELISA and verified by qRT-PCR. | 1, 10, 50, 100, 250, 500 ug/ul (DNA fragemntation)  10, 100, 500, and 1000μg/ml | Glyceraldehyde-3-phosphate dehydrogenase (GAPDH) was used as a positive control | 24, 48, 72 hrs | *T. serpyllum* extract induced cytotoxicity in breast cancer cells (MCF-7 and MDA-MB-231) but not in normal cells. It also induced apoptosis and inhibited the DNMT and HDAC activities in MDA-MB-231 cells. | (Bozkurt et al., 2012) |
| Antimicrobial and antifungal activity | T. serpyllum, 4 compounds isolated from T. serpyllum were tested | In vitro  Agar diffusion assay | 0.05 mg (50 L of 1 mg/mL) | Penicillin  Tetracycline  Nystatin  Actidione |  | *n*-Propyl Rosmarinate showed the most potent activity against both bacteria. The antifungal  activity of the pure compounds was tested against two microorganisms, and *n*-Propyl Rosmarinate was found to be resistant to *Aspergillus niger.* | (Aziz et al., 2014) |
| Antimicrobial activity | Ethanolic extract (70%) of T. serpyllum | In vitro  Microdilution method using Mueller–Hinton (MH) broth and MH broth with 5% lysed sheep/horse blood, or MH broth with 2% glucose for growth of fungi. Minimal Inhibitory Concentration, Minimal bactericidal concentration or minimal fungicidal concentration | 20 to 0.156 mg/mL | DMSO control (at a final concentration of 10%), a positive control (containing inoculum without the tested extracts)  Negative control (containing the tested extracts without inoculum) on each microplate | 24 hrs except for h. pylori 72 hrs | The ultrasonic extracts showed a wide spectrum of antimicrobial activity, the bactericidal or bacteriostatic activity against tested bacteria and fungi at a concentration of 0.0625–20 mg/ml, but there were differences in their strength of action against test strains of microorganisms. | (Ivasenko et al., 2021a) |
| Antimicrobial activity | Essential oil of *T serpyllum* | In vitro  Agar diffusion test | 20 ul | Positive controls: gentamicin (10 mg/disk) for Gram-negative bacteria, ampicillin (10 mg/disk) for  Gram-positive bacteria, and nystatin (100 UI) for Candida. Negative control: DMSO |  | Wide spectrum of antimicrobial activity was recorded in essential oil samples of *T. Pullegoides* and *T. serpyllum*, which have an aromatic-monoterpenoid chemotype. This will suggest its important use in the pharmaceutical industry, especially in food industry and as preservative. | (Kryvtsova et al., 2022) |
| Antimicrobial activity | Ethanolic extract of *T serpyllum* | In vitro  Minimum inhibitory concertation, microdilution and bacterial inhibition rate methods | 15mg/ml to 60mg/ml |  |  | *T. serpyllum* showed good antimicrobial activity with an inhibition rate of 132.93% against E. coli and 78.40% against C. albicans. | (Horablaga et al., 2023) |
| Antibacterial activity | Essential oil of *T serpyllum* | In vitro  Minimum inhibitory concertation, and serial dilution methods | 125ug/ml to 1000ug/ml |  |  | The bactericidal minimal inhibitory concentrations (MICs) of essential oil of *T. serpyllum* were determined for all strains of  staphylococci (for 5 – 1097,5, for 1 – 2195 μg/ml). The bacteriostatic MICs were determined for 3 strains (for 2 – 548,75, for 1 – 1097,5 μg/ml). MIC50 of *T. marschallianus and T. serpyllum* for the test staphylococcal strains amounted 108,89 and 496,59, for the test strains of gram-negative bacteria 683,91 and 783,43 μg/ml respectively. The antistaphylococcal activity of the essential oils of *T. marschallianus* was significantly higher than this activity of the essential oils of *T. serpyllum*. | (Shapoval et al., 2023) |
| LDL levels and oxidations | Essential oils and aqueous tea infusions of oregano (Origanum vulgare L. spp. hirtum), thyme (Thymus vulgaris L.) and wild thyme (Thymus serpyllum L.) | In vitro  copper-induced oxidation of human low-density lipoproteins | 0.002g/l, 0.004g/l, 0.02g/l for EO, and 0.0002g/l, 0.02g/l, 0.24g/l for aqueous tea infusion |  | 30-1200 minutes | LDL samples enriched with 0.004 g/l of essential oil exhibit a prolonged lag phase (40 min for oregano, thyme and wild thyme essential oils) as compared with the native LDL sample (10 min). LDL samples enriched with 0.02 g/l of essential oil also exhibit a prolonged lag phase (110 min for thyme) in comparison with native LDL (30 min). LDL samples exposed to 0.02 g/l essential oils of oregano and wild thyme never entered a propagation phase of oxidation. LDL samples enriched with 0.002 g/l applied essential oils did not have effect to the kinetics of LDL oxidation. The kinetics of copper-induced LDL oxidation in the presence of 0.02 g/l aqueous tea infusions of oregano, thyme and wild thyme. The presence of thyme and wild thyme aqueous tea infusions leads to the prolongation of the lag phase of LDL oxidation from 30 min for native LDL to 250 min and 1200 min for LDL exposed to thyme and wild thyme, respectively. | (Kulisic et al., 2007) |
| Antioxidant and Anticholinesterase activity | Ethanolic extract of T. serpyllum subsp. serpyllum | In vitro  AChE inhibitors Ellman’s colorimetric method | 0.25, 0.5 and  1mg/mL | Galantamine  (0.03–1 𝜇g/mL |  | *T. serpyllum* did not show potent inhibitory activity against AChE with IC50 value *T. serpyllum* subsp. *Serpyllum* (IC50 = 742.11 𝜇g/mL). | (Kindl et al., 2015) |
| Antimicrobial activity of polyherbal/Oral health | Poly herbal mixure of Salvia officinalis, Rosemaryinus officinalis, Thymus serpyllum, Cinnamomum zeylanicum, Mentha arvensis | In vitro  Agar diffusion method | 25μL 50μL,100μL | Amoxicillin |  | At 50μl and 100μl, the antimicrobial activity against *S. aureus* was found to be statistically significant when compared to the standard (p<0.05). At 25μl and 100μl, the antimicrobial activity against *E. faecalis* was found to be statistically significant when compared to the standard. | (Kavyashree and Rajasekar, 2021) |
| Antimicrobial activity | Ethanolic, decoction, and infused extracts of T. serpyllum L | In vitro  Agar disk diffusion method | 2.5 mg/ml | Antibiotics including Imipénème 10 ug/ml, Ceftazidime 30 ug/ml, Gentamicine 10 ug/ml, Chloramphénicol 30 ug/ml, Acide nalidixique 30 ug/ml Aztréonam 30 ug/ml. Antifungal including Fluconazole 10,20,50, 100 ug/ml, Amphotéricine 10, 20, 50, 100 ug/ml and Terbinafine 10, 20, 50, 100 ug/ml. |  | The ethanolic extract of *T. serpyllum* presented a good antimicrobial activity against all the tested strains with the inhibition diameters from 7 to 28 mm. The decoction extract inhibited the growth of *E. coli, C. albicans*, and *Aspergillus brasiliensis* with the inhibition diameters between 11 to 20 mm. | (Tamert et al., 2017) |
| Bactericidal for GIT disorders against H. pylori | Aqueous ethnaolic extract | In vitro  MBC | Final concentrations of each extract were  500, 250, 125, 62.5, 31.2, 15.6, 7.8_g/ml in the mixture. | Amoxicillin as a positive control and DMSO as a negative  control |  | *T. serpyllum* extract including other plant extract completely inhibited the growth of *Helicobacter pylori* at 500 g/ml in all strains. | (Zaidi et al., 2009) |
| Alpha glucosidase inhibitor | Aqueous and methanolic extract | In vitro  Alpha glucosidase inhibitors |  | 20 ul Acarbose (100 ug) |  | Aqueous extract of *T. serpyllum* showed 50-75% inhibitory effect of alpha glucosidase. | (Gholamhoseinian et al., 2008) |
| Cytotoxicity/Breast cancer | Aqueous extracts of T. serpyllum, Thymus vulgaris | In vitro  MTT assay  MCF-7/wt and MCF-7/Adr cells | 8, 16, 32, 63, 125, 250, 500, 1000, 2000 mg/l of dried aqueous extracts | MCF-7/wt or MCF-7/Adr cells treated in an analogical mode, incubated in media complemented with distilled water instead  of the solution containing the tested substances |  | Two thyme extracts exhibited a comparable effect on MCF-7/Adr cells reaching EC50 values around 400 mg/l, which was in-between the results obtained for ExMp (with nearly 2-fold lower toxicity) and ExMh (showing nearly 2-fold higher toxicity). | (Berdowska et al., 2013) |
| Antithrombin activity | Methlene chloride, Methanolic extract of *T. serpyllum* | In vitro  Antithrombin assay and cytotoxicity 9 mouse leukemia cells | 50 ul | Methotrexate |  | Anticancer activity in the methylene chloride extract of the *Quercus robur*, P. major and *T. serpyllum* plants tested is similar of that of the methanol extract. | (Goun et al., 2002) |
| Antibacterial activity | Ethanolic extract | In vitro  Agar well diffusion, disk diffusion, MIC | 10 ul | Penicillin G (10 units), Nalidixic  acid (30 μg), Vancomycin (30 μg), Tetracycline (30 μg), Chloramphenicol  (30 μg), Novobiocin (5 μg), Ampicillin (5 and 30 μg) as positive control and ethanol as a negative  Control |  | Clove and wild thyme extracts were found to be equally effective against both gram-positive and gram-negative. | (Bayoub et al., 2010) |
| Microsomal Lipid peroxidation/liver injury/hepatoprotective | PC-1) containing or | In vitro  HepG2 human hepatocellular carcinoma cell line  Transient transfection and luciferase reporter gene assay, microsomal LPO, and osmotic resistance  of erythrocytes  In vivo  Rats intoxicated by carbon tetrachloride. | 200-2000 μg/ml  (100-200 mg/kg (in vivo) | tert-butylhydroquinone (tBHQ)  100 mg/kg α-tocopherol (in vivo) |  | Formulation containing *O. vulgare, T. serpyllum*, *T. cordata, T. farfara* and *V. officinalis* exhibited more pronounced LPO-inhibitory and antihemolytic effects in vitro than its individual components. The formulated extract activated the Nrf2/antioxidant response element signalling pathway more effectively than the extracts of other phytocompositions. Oral administration of formulated extract markedly ameliorated liver injury in rats acutely or chronically intoxicated by carbon tetrachloride. | (Aralbaeva et al., 2017) |
| Antigenotoxicity effect/Antidiabetic activity | Ethanolic, methanolic, aqueous extract of T. serpyllum | In vitro  α-glucosidase inhibition assay | 0.1 mg/mL, 0.25 mg/mL, and 0.5 mg/mL) | Acarbose |  | Aqueous extracts of T. serpyllum, M. piperita, S. montana, and O. vulgare at 0.5 mg/mL showed the highest inhibitory activity against yeast α-glucosidase enzyme, with corresponding inhibition percentages of 98.98 %, 92.62 %, 89.38 %, and 78.40 %, respectively. These extracts exhibited significantly better activities than the one shown by the positive control (acarbose), inhibiting 71.35 % of α-glucosidase activity at the same concentration (0.5 mg/mL).  At lower concentration (0.25 mg/mL), aqueous extracts of T. serpyllum, M. piperita, S. montana exhibited 96.73 %, 81.53 %, and 72.11 % α-glucosidase inhibitory activity, again all significantly higher compared with acarbose (58.06 %) at the same concentration.  At the lowest extract concentration applied (0.1 mg/mL), aqueous extracts of T. serpyllum and M. piperita demonstrated significantly higher percentages of α-glucosidase inhibitory activity (88.07 % and 53.61 %) as compared with acarbose (36.76 %). At the same concentration, **E-**extracts of M. officinalis and T. serpyllum have also shown significantly better activities than the positive standard (44.34 % and 44.24 % for M. officinalis, and T. serpyllum vs. 36.76 % for acarbose). | (Pavlović et al., 2022) |
| Antimicrobial activity | Essential oils and chloroformic extracts of T. serpyllum | In vitro  Agar well diffusion method | 15, 10 and 5μl of essentail oil, and  100, 200 and 300  μg/cm³) for the chloroform extract | Ciprofloxacin dissolved in DMSO at 100  μg/cm3 as positive control, and sterile DMSO as negative control. |  | The essential oils and chloroformic extracts of *T. serpyllum* were effective against the tested strains of bacteria. | (Abu-Darwish et al., 2012) |
| Antibacterial activity | Mixture design of Myrtus communis, Artemisia herba-alba and Thymus serpyllum essential oils | In vitro  Microdilution, and Agar Disk-diffusion assays  MIC, MBC | 10 ul |  | 18-24 hrs | *T. serpyllum* alone ensured the optimal inhibition against *S. aureus* and *E. coli*, while a ternary mixture consisting of 17.1%, 39.6% and 43.1% of *M. communis*, A. herba-alba  and *T. serpyllum* respectively, was associated with optimal inhibitory activity against *B. subtilis.* *T. serpyllum* demonstrated the most marked antibacterial effect with a MIC and MBC from 0.125 to 0.5%. | (Ouedrhiri et al., 2022) |
| Antimicrobial activity | T. serpyllum essential oil | In vitro  Agar diffusion assay | 10 ul | Positive controls: gentamicin (10 mcg/disk); ampicilin  (10 mcg/disk), amoxicillin/clavulanic acid (20/10 mcg/disk);  nystatin (100 UI) and itraconazol (30 mcg/disk) for Candida strains.  Dimethyl sulfoxide (DMSO) as a negative control. | 24-48 hrs | *T. serpyllum* essential oil displayed antibacterial properties against a wide spectrum of the microorganisms. The antimicrobial properties could be attributed to the high content of aromatic monoterpenoid thymol (47.33%). | (Shanaida et al., 2021) |
| Antibacterial and antifungal activity | T. serpyllum essential oil | In vitro  Agar well diffusion and broth dilution assays | 10 ul | DMSO (10%, v/v) as negative control in both the assays, and antibiotics as vancomycin, tetracycline, fluconazole and amphoteric as a positive control against bacterial and fungal strains, respectively | 24-48 hrs | Antimicrobial activity as zone of inhibition (ZOI) varied from 13.66 ±0.58 mm to33.66 ±1.52 mm, while thymol (10%, v/v) showed ZOI ranged from 15.5 ± 0.5 mm to 26.33 ±2.08 mm against tested bacterial and fungal species. MIC was 0.039% to 0.078% against tested bacterial and fungal species, whereas thymol showed 1.25% to 2.5% MIC against tested bacterial and fungal species. Different combinations of EO (2MIC to1=2MIC) and thymol (2MIC to1=2MIC) with antibacterial and antifungal antibiotics (2MIC to1=2MIC) were found to increase the efficacy of antibiotics by 4-130 folds against bacterial and fungal pathogens. | (Salaria et al., 2022) |
| Antibacterial activity | *T. serpyllum* essential oil | In vitro  Minimum inhibitory concentration and minimum bactericidal concentration | 0-0.35 ug/ml with 1Mm CA or 3% N  aCL |  |  | Supplementation of *T. serpyllum* essential oil with Caprylic acid, the value of MIC reduced by  29%, while enrichment of essential oil with 3% NaCl, the MIC value reduced by 63% (from 0.209-0.046 to 0.077-0.018 L/mL). Most of the non-supplemented essential oil showed lower activity against MDR and ESBL producing *E. coli,* while supplemented essential oils with caprylic acid and sodium chloride were effective against ESBL and non-ESBL as well as MDR and non-MDR E. coli. The essential oils supplementation with NaCl could enhance the antibacterial activity towards ESBL and MDR *E. coli* isolates. | (Gāliņa et al., 2022) |
| Antifungal activity | *T. serpyllum* essential oil | In vitro  mycelial growth inhibition | 0.25, 0.5, 1, 5, 10 and 15 mg/L and 0 for control | Thiophanate-methyl |  | The mycelium is less affected in the presence of *T. serpyllum essential oil* at the 0.25 mg/L, MGI has a value of 99%. At 0.5 mg/L *T. serpyllum essential oil* the MGI value drops at 22%. | (Rus et al., 2015) |
| Antioxidant and anticandidal activity | *T. serpyllum* essential oil | In vitro  Disk Diffusionand MIC, MCC | 10 ml of oil/disc | 40 mg of  fluconazole/disc. |  | *T. serpyllum* essential oil did not show potent anticandidal/antifungal activity compared to other essential oils tested. | (Jamali et al., 2012) |
| Rickettsia slovaca and Rickettsia conorii capsia | *T. serpyllum* essential oil | In vitro  Vero cell lines | 0.01% |  |  | Wild thyme oil may inhibit the growth of rickettsiae in vitro experimental models. | (Štefanidesová et al., 2018) |
| Antifungal activity (skin) | *T. serpyllum* essential oil | In vitro  MIC values and MFC (minimum fungicide concentration) | 0.039-10 mg/ml (MIC)  10–0.5 mg/ml (MFC) | Ketoconazole |  | Cinnamon bark oil, thyme oil, clove oil, geranium oil and manuka oil have the strongest antifungal properties. The minimum inhibitory concentration (MIC) values of the fungi species in the tested oils were in the range of 0.5- > 10 mg/ml and the minimum fungicidal concentrations (MFC) in the range of 1.25- > 10 mg/ml. Cinnamon oil and thyme oil showed the strongest effect on test fungi. The strongest activity of these oils was observed against dermatophyte fungi. | (Michalczyk and Ostrowska, 2021) |
| Antioxidant and photoprotective (skin protective) activity | Essentail oil, ethanolic and infusion extracts of T. serpyllum |  |  |  |  | Ethanolic and infusion extracts exhibited high values of Sun Protective Factors (SPF) with 38.34  ± 2.29 and 38.82 ± 2.23 for ethanol and infusion extract, respectively. These results suggest a potential use of *T. serpyllum* as a source of bioactive compounds with antioxidant and skin-protective properties. | (Madouni et al., 2021) |
| Antifungal activity | T. serpyllum essentail oil | In vitro  MIC, MFC |  | Nystatin and chlorhexidine |  | The antifungal activity against oral isolates of *C. albicans* and *C. glabrata* was found as follows: thyme essential oil (MIC = 125 – 500 mg/L) ≥ oregano essential oil (MIC = 250 – 500 mg/L) = summer savory essential oil (MIC = 250 – 500 mg/L) ≥ wild thyme essential oil (MIC = 500 – 1000 mg/L). | (Baj et al., 2020) |
| Antimicrobial synergetic activity of 3 essential oils | Lavandula dentata/Origanum  majorana EOs and T. serpyllum/Origanum majorana essentail oils | In vitro  Agar disk diffusion, MIC, MBC | 4%, 2%, 1%, 0.5%, 0.25%, 0.125%, 0.0625%, 0.03125%, and 0.01562% (v/v). |  |  | Strong inhibitory effect of *O. majorana* (MIC: 0.125% v/v) and *T. serpyllum* (MIC: 0.0625; 0.125 % v/v) EOs against E. coli and S. aureus. It can be also noted that *T. serpyllum* EO exhibited the highest antibacterial effect against E. coli with MIC value four-fold lower compared to L. dentata EO and two-fold lower compared to O. majorana EO. T. serpyllum EO exhibited the strongest bactericidal effect against both tested strains.  The combined effect of *O. majorana* and T. serpyllum essential oils showed that all tested combinations displayed a partial synergistic effect against both studied strains with a FIC index of 0.75. Two combinations of, respectively, 1/4 MIC marjoram + ½ MIC thyme and 1/2 MIC marjoram + 1/4 MIC thyme were found antibacterial against S. aureus. One combination of 1/2 MIC marjoram + 1/4MIC thyme showed an antibacterial effect against E. coli. | (Ouedrhiri et al., 2017) |
| Antimicrobial activity | *T. serpyllum* essential oil | In vitro  Agar well diffusion, MIC, MBC | 25%, 12.5%, 6.25%,  3.13%, 1.56%, 0.78%, 0.39%, 0.20%, 0.10%, 0.05% to 0.025%  and 0.012% v/v in final volume | Control wells contained broth and broth with bacteria were used, respectively, as negative or sterility control and  positive control. |  | The most beneficial effect were *O. vulgare* (MIC range 0.012%-0.2%, mean 0.045%; MBC range 0.012%-0.2%, mean 0.049) and *T. serpyllum* (MIC range 0.012%-0.2%, mean 0.056%; MBC range 0.012%-0.2%, mean 0.062%), *M. alternifolia* (MIC range 0.10%-0.2%, mean 0.2%; MBC 0.2%, mean 0.2%), and *T. vulgaris* (MIC range 0.05%-0.39%, mean 0.14%; MBC range 0.05%-0.39%, mean 0.15%) showed a moderate effects against tested strains; the results are considered statistically significant (*P-*value <0.01). | (Listorti et al., 2020) |
| Antimicrobial activity | *T. serpyllum* essential oils | In vitro  Agar dllution, and MIC | 0.125 to 0.500 mg/mL. | chlorhexidine gluconatc |  | T herba barona oils showed antimicrobial properties comparable to those of *T. vulgaris* and *T. serpyllum* essential oils. Strain of P*s. aeruginosa* results slightly more sensitive to these latter oils (MIC., 0.5 mg/mL) than to T. herba barona oils (MIC., 2.000 and 1.000 mg/mL, respectively). | (Juliano et al., 2000) |
| Antibacterial activity | *T. serpyllum* essential oil in vapour phase | In vitro  Micro‐atmosphere and disk diffusion assay | 32, 16, 8, 4, 2, 1, 0.5, 0.25 μl) | Vancomycin 30 μg,  erythromycin 15 μg, and oxacillin 1 μg. |  | The most active was Armoracia rusticana with MICs ranging from8.3 to 17 μl/l,  followed by Origanum syriacum (8.3–130 μl/l), Allium sativum (8.3–530 μl/l), Satureja hortensis (17–130 μl/l), Satureja montana (33–260 μl/l), *T. vulgaris* (33–260 μl/l), and *T. serpyllum* (33–530 μl/l). | (Nedorostova et al., 2011) |
| Antibacterial activity | *T. serpyllum* essential oil | In vitro  Microdilution, MBC and MIC | 50 to 0.2 μl/ml |  |  | Wild thyme oil was efficient in the following range of concentration MIC/MBC=3.125-6.25/6.25-12.5 μl/ml for Gram-positive bacteria used, while it only showed an effect at concentrations MIC/MBC=1.56/3.125 μl/ml for Gram-negative bacterial strains. The strongest antibacterial effect was shown by oregano essential oil, while the oil extracted from wild thyme was least potent. The antibacterial activity of essential oils declined with decreasing concentration, regardless of the tested bacteria. | (Jovanka et al., 2011) |
| Antibacterial activity | Primary  and secondary essential oils | In vitro  ZOI, MIC | 5 ul |  |  | The secondary essential oils of *T.serpyllum* and *T. linearis* were rich in phenolic compounds (92.3 % and 96.4 %, respectively) as compared to  primary essential oils (42.1 % and 47.9 %, respectively). The bioassay showed that the secondary oils of both the *Thymus* spp. exhibited stronger antibacterial and antifungal activities than primary oils. | (Verma et al., 2016) |
| Antitumor/antimicrobial activity (Oral care/Oral pathogen) | T. serpyllum essential oil | In vitro  MIC/MBC  MCF-7 (breast adenocarcinoma), NCI-H460 (non-small cell lung cancer), HCT-15 (coloncarcinoma), HeLa (cervical carcinoma), and HepG2 (hepatocellularcarcinoma) | >400 g/mL | Ampicillin and Streptomycin), mycotic (Fluconazole), and acommercial antimicrobial mouth rinse Hexoral®  100 U/mL penicillin and 100 g/mL streptomycin. Ellipticinewas positive control (0.24–65.2 g/mL) |  | T. serpyllum oil showed the strongest activity against both type of microorganisms (MIC 2.5–5 g/mL and MBC 5–10 g/mL for bacteria and MIC 1–2 g/mL and MFC 2–4 g/mL for fungi). T. serpyllum oil was the most potent in all tested cell lines, presenting GI50 values ranging from 7.02–52.69 g/mL. | (Nikolic et al., 2014) |
