# Supplementary material for: Wild thyme (Thymus serpyllum L.): a review of the current evidence of nutritional and preventive health benefits*
Source: Front Nutr. 2024 May 23;11:1380962. doi: 10.3389/fnut.2024.1380962 (PMC11153689; doi:10.3389/fnut.2024.1380962)
Supplement: Supplementary file 3 [file Table_3.docx]

| **Table S3** Summary of the reported pre-clinical (In-vivo studies) for *Thymus sepyllum* essential oils and extracts (n=12) | | | | | | | |
| --- | --- | --- | --- | --- | --- | --- | --- |
| **Properties investigated** | **Extract** | **Model** | **Dose range tested** | **Control used** | **Duration of study** | **Outcome** | **References** |
| Intestinal anti-inflammatory activity | Aqueous extract of T. serpyllum | In vivo  Trinitrobenzenesulfonic acid (TNBS) model of rat colitis  Dextran sodium sulfate (DSS) model of mouse colitis | 10, 100, 250 and 1000 mg/kg | Sulphasalazine at the dose of 30 mg/kg. It was suspended in 1 mL of  carboxymethylcellulose (0.2%) and Tween 80 (1%) (CMC) in  water solution and administered daily by oral gavage. The  untreated TNBS control group and a non-colitic group were  also included for reference, which received orally the solution  used to administer the test compounds (1 mL).  The remaining group (colitic control mice) was given the vehicle (200 μL of CMC) | The different doses of T. serpyllum extract, or sulphasalazine, were administered from two days before colitis induction and, therefore, daily until the day before the sacrifice of the rats, i.e. eight days after TNBS administration. | *T. serpyllum* extract showed intestinal anti-inflammatory effects in experimental models of colitis, as reported histologically, since it facilitated the tissue recovery of the damaged colon and biochemically since it improved the different inflammatory markers evaluated, including myeloperoxidase activity, glutathione content, and leukotriene B_4_ levels as well as the expression of the inducible proteins iNOS, and COX-2. This effect was associated with the reduction in the expression of different cytokines, like TNFa, IL-IB, IFNy, IL-6 and IL-17, the chemokine MCP-1, and the adhesion molecule ICAM-1, hence ameliorating the altered immune response associated with the colonic inflammation. | (Algieri et al., 2014) |
| Protective activity against toxicity to the liver and the inflammation of intestines | The mixture of commercial lactobacteria Lactobacillus helveticus with a water-soluble extract of thyme (T. Serpyllum L.) on a sterile milk basis. | In vivo  White rats with intestinal dysbacteriosis due to carbon tetrachloride (CCl4) and ampicillin trihydrate. | 150 mg/kg |  | 21 days | For toxic liver damage by CCl4 on days 7, 14, and 21.  Analysis of the effects of the phytobacterial agent on  the liver and ileum damaged by CCl4 revealed that the  phytobacterial agent improved the microbiological  indicators of the intestinal flora and the morphological  parameters of the liver in the experimental animals. | (Tarmakova et al., 2019) |
| Anti-inflammatory activity | Codelac broncho elixir | In vivo  Rats | Injected to rats intragastrically in a volume of 0.17 ml 4 times a day. | Fenspiride  (Erispirus syrup, 2 mg/ml, SANDOZ) was injected  intragastrically in a volume of 0.29 ml 4 times a day. | 120 hrs | In case of injecting Codelac® Broncho with T. Serpyllum, there was a significant decrease in the paw volume increment compared to that in the control group,  starting with 4 hours after the initiation of edema, by 42.4% (*p*<0.05), while in the group with the injection of the comparison drug, there were no significant differences observed from the values of the control group. In the group with the injection Fenspiride, 8 hours after the induction of edema, there was observed a significant decrease in the paw volume increments in comparison with the control group – by 40.6% (*p*<0.05), while in the group with the injection of the studied drug there were not significant differences observed from the control. Anti-inflammatory activity of Codelac® Broncho with  T. Serpyllum in comparison with Fenspiride, on the model of acute carrageenan inflammation of the paw in rats. | (Kolesnichenko et al., 2019) |
| Burns (second and third degree) | Robacin (Rosa damacena, Calendula officinlis, and beeswax  Topical aloe vera: topical Rimojen (mixture of thymus serpyllum, macrophyllum and platony-chium | In vivo  Rats Responses to the treatment were assessed by digital photography during the treatment until day 32. Histological parameters (PMN, epithelialization, fibrosis, and angiogenesis) |  | Topical silver sulfadiazine | 32 days | The wound had better healing in Robacin group. Also, speed of healing was better in aloe vera group than silver sulfadiazine and Rimojen groups. In terms of wound surface area maximal improvement was observed at the same time in the second and third degree burn wounds in Robacin group, in the second degree wound of aloe vera and Rimojen groups, and in the third degree wound of aloe vera and silver sulfadiazine groups. | (Akhoondinasab et al., 2015) |
| Membrane stabilising properties/LPO/Liver | Aqueous ethanolic extract of *T. serpyllum* | In vivo  Wistar rat model Lipid peroxidation (LPO) in liver microsomes | 0 to 200 _g dry substance/mL |  |  | *T. serpyllum* extract exhibited a higher antioxidant activity (IC50 = 3.3 _ 0.7) compared to other plant extracts. Accordingly, among the extracts studied, those from Salvia officinalis, *T. serpyllum*, and Origanum vulgare show the most pronounced membrane-stabilizing activity. | (Ydyrys et al., 2021) |
| Hypertension /Cardiovascular | Aqueous extract of T.serpyllum | In vivo  Hypertensive and normotensive W rats | I.V  bolus of extract 100mg/kg b.w dissolved in 0.2ml saline | 0.2ml saline |  | Wild thyme induces a decrease of blood pressure and vascular resistance in hypertensive rats. The inverse correlation between vascular resistance and plasma heme oxygenase-1 suggests that endogenous vasodilator carbon monoxide generated by heme oxidation could account for this normalization of blood pressure. | (Mihailovic-Stanojevic et al., 2016) |
| Antidiabetic activity | Aqueous extract of T.serpyllum | In vivo  Diabetic rabbits | 500mg/kg b.w | Glibenclamide and acarbose | 3 months | Ether and aqueous extracts significantly reduced the blood glucose level with maximum effect (p<0.001) produced by aqueous extract. Aqueous extract significantly inhibited the rise in glucose level in oral glucose tolerance test. The extract showed synergistic effect with different doses of insulin; while, serum insulin level of the diabetic rabbits was not significantly increased by the extract. HbA1c level was significantly (p<0.05) reduced whereas hemoglobin level was significantly increased in three months study. | (Alamgeer and Mushtaq, 2017) |
| Lipid profile/Liver enzymes | Aqueous extract of T.serpyllum | In vivo  Diabetic rabbits | 500mg/kg b.w | Glibenclamide and acarbose | 30 days | Wild thyme extracts significantly reduced the level of serum cholesterol, triglyceride, LDLs, VLDLs, alkaline phosphatase and transaminases without affecting the HDLs level. Total cholesterol/HDL-cholesterol ratio was significantly reduced as compared to diabetic control. | (Alamgeer et al., 2016) |
| Antioxidant and antihypertensive activity | Aqueous extract of T.  serpyllum L. | In vivo  In spontaneously hypertensive rats (SHR) and in normotensive Wistar rats. | 0, 10, 50, and 100 mg/kg  b.w. (dissolved in 0.2ml saline) |  |  | Bolus injection of the extract (100mg/kg body weight i.v.) induced significant decrease of systolic and diastolic blood pressure and total peripheral resistance in SHR, without effects on these parameters in normotensive Wistar rats. Cardiac index remained unchanged after treatment in all experimental rats. | (Mihailovic-Stanojevic et al., 2013) |
| The impact of its antioxidant activity on the inflammatory status and gut dysbiosis. | Standardization on 70% native extract (DER native 4-8:1) and 30% Dextrin. | In vivo  high-fat diet-induced obesity mice | 50, 100 and 150 mg/kg in High-fat diet-fed mice | Control diet  treated with thyme extract at 150 mg/kg | 10 weeks | Mice that consumed the High-fat diet exhibited TBARS values significantly higher than both standard diet groups. Conversely, the administration of thyme extract in HFD-fed mice was able to significantly reduce the TBARS values. The daily administration of thyme extract to HFD-fed mice significantly reduced weight gain from day 6 onwards at all doses assayed, even though food intake was similar in all HFD-fed groups throughout the experimental period. | (Ruiz-Malagon et al., 2022) |
| Antidiabetic activity | Silver nanoparticles using aqueous extract of T. serpyllum | In vivo  Diabetic mice (Streptozotocin-Induced Diabetic BALB/c Mice) | 5 mg/kg, and 10mg/kg | Metformin at 100 mg/kg | 4 weeks | Silver nanoparticles at 10 mg/kg could increase in the expression of AMPK and IRS1, hence, increasing the glucose uptake in cells. | (Wahab et al., 2022) |
| Antidiabetic activity | Aqueous extract of T.  serpyllum L | In vivo  Diabetic mice (Streptozotocin-Induced Diabetes Mellitus Type 2 Mice) | 500 mg/kg/d and 800 mg/kg/d) | Metformin at 100 mg/kg | 4 weeks | Aqueous extract of T. serpyllum was found to be effective in controlling hyperglycemia and improving glucose and insulin tolerance. This impact was attributed to the upregulation of the AMPK expression at  the mRNA level, and upregulating the expression of IRS1 and GLUT2 gene. The liver, kidney, and pancreas histological examination showed the restorative impact in terms of cellular morphology. | (Azhar et al., 2022) |
